# Porphyrin-Based Nanomaterials for the Photocatalytic Remediation of Wastewater: Recent Advances and Perspectives

**DOI:** 10.3390/molecules29030611

**Published:** 2024-01-26

**Authors:** Nirmal Kumar Shee, Hee-Joon Kim

**Affiliations:** Department of Chemistry and Bioscience, Kumoh National Institute of Technology, Gumi 39177, Republic of Korea; nirmalshee@gmail.com

**Keywords:** porphyrin, self-assembly, nanomaterial, toxic pollutant, photocatalysis

## Abstract

Self-organized, well-defined porphyrin-based nanostructures with controllable sizes and morphologies are in high demand for the photodegradation of hazardous contaminants under sunlight. From this perspective, this review summarizes the development progress in the fabrication of porphyrin-based nanostructures by changing their synthetic strategies and designs. Porphyrin-based nanostructures can be fabricated using several methods, including ionic self-assembly, metal–ligand coordination, reprecipitation, and surfactant-assisted methods. The synthetic utility of porphyrins permits the organization of porphyrin building blocks into nanostructures, which can remarkably improve their light-harvesting properties and photostability. The tunable functionalization and distinctive structures of porphyrin nanomaterials trigger the junction of the charge-transfer mechanism and facilitate the photodegradation of pollutant dyes. Finally, porphyrin nanomaterials or porphyrin/metal nanohybrids are explored to amplify their photocatalytic efficiency.

## 1. Introduction

Environmental pollution has become a significant issue. Earth suffers from pollution (air and water) resulting from the rapid development of civilization and industrialization. Every year, large quantities of dangerous compounds, including synthetic dyes, cosmetics, insecticides, pharmaceutical products, and aromatic compounds, are released from the textile, leather, drug, and paper printing industries into water. These toxic pollutants not only damage the potability of water but also pose a significant risk to the entire ecosystem and have detrimental impacts on marine life [1,2]. This has led to an increasing number of scientific reports by environmentalists and researchers on developing strategies for the environmental purification of wastewater [3,4,5,6]. Various physicochemical methods, including filtration [7], adsorption [8], precipitation [9], chemical coagulation [10], electrochemical methods [11], bacterial treatment [12], and advanced oxidation processes (AOPs) [13], have been demonstrated in previous reports to remove these toxic pollutants from contaminated water. AOPs are the most adaptable techniques used in pollution control because of their uncomplicated procedures, economic affordability, and high activity for the degradation of hazardous pollutants to less hazardous H_2_O and CO_2_ without the creation of any other secondary pollutants. In AOPs, a suitable photocatalyst absorbs visible light and generates ROS in situ, thereby accelerating the removal of organic compounds from water. In particular, photocatalysis has received considerable attention, as it benefits from ecological advantages and additional economic benefits related to the utilization of freely accessible natural sunlight. Moreover, its mechanism is simple. First, light harvesting followed by exciton diffusion occurs, and after that, charge separation takes place, which is accompanied by carrier transport. Therefore, light absorption and successive electron shifts are pivotal for achieving efficient solar energy harvesting using photocatalysts. Nanoscale architectures in photofunctional compounds traditionally display typical optoelectronic properties based on their sizes and shapes. Consequently, the fabrication and design of nanoscale assemblies are important for constructing photocatalytic systems with specific properties and functions [14,15].

Considering the superiority of photocatalytic processes, several organic and inorganic-based nano- and micro-structures have emerged as new building blocks to fabricate photocatalytic systems. Various inorganic nanostructures, including metal oxide nanoparticles (MNPs) (TiO_2_ [16,17] and ZnO [18,19]), graphitic carbon nitride (g-C_3_N_4_) [20,21], silicates [22,23], zeolites [24,25], and bismuth-based photocatalysts [26,27], have been used to eliminate hazardous pollutants from wastewater. Titanium dioxide (TiO_2_) has been used as an emerging catalyst because of its high stability, moderate toxicity, economic affordability, and excellent catalytic properties for the decay of toxic contaminants in aqueous solutions. However, its high E_g_~3.2 eV arising from the high strength of recombination between photogenerated pairs (hole = *h*^+^ and electron = *e*^−^) limits its absorption of solar light (λ < 370 nm). This leads to a low photodegradation efficiency under visible light. It is extremely difficult to use these catalysts multiple times, and their complete recovery at the end of the reactions that they catalyze is impossible. Moreover, a large quantity of photocatalysts is required for loading in the photocatalytic method to achieve a reasonable degradation rate [28,29].

Conversely, organic nanostructures have secured unusual recognition compared with their inorganic analogs. This is because of the substantial versatility of their molecular schemes and the tremendous adjustability of their photoelectronic and solution-phase properties, which make them suitable for photocatalytic systems. Among the several organic building blocks, such as carbon quantum dots [30], fullerene [31], and graphene oxide or reduced graphene oxide [32], used to construct nano- and micro-structured organic nanomaterials with well-defined shapes and dimensions, the distinguished π-conjugated planar porphyrinoids (free porphyrin or metalloporphyrin) are of great interest for their tremendous optoelectronic properties and variable coordination features [33,34,35,36]. Owing to their large absorption coefficients in the Soret (400–440 nm) and Q bands (500–720 nm), porphyrin compounds are efficient at capturing light over a longer range (UV–vis) and initiating ROS across a spin-forbidden intersystem crossing pathway. Additionally, their rigid structural framework and intrinsic aromatic electronic qualities initiate self-aggregation in the solid and solution phases. However, the use of free or immobilized porphyrin compounds in photocatalysis is restricted because of their easy agglomeration in solutions and their deactivation, lower reusability, and difficulties in the separation of catalysts after the reaction. Therefore, new photocatalysts must be developed to overcome these challenges.

For this purpose, porphyrin-based nanomaterials have emerged as photocatalysts for the decay of hazardous pollutants in wastewater over the last two decades, and they are highly regarded by material chemistry researchers [37,38,39,40]. After aggregation, the formation of a nanoassembly of porphyrins not only increases solar absorption but also enhances stability for recycling. Moreover, their rigid framework resists hydrolysis and increases their photostability for further use. Numerous intermolecular non-covalent interactions (π-π stacking interactions; hydrogen bonding interactions; electrostatic, hydrophobic, and hydrophilic interactions; and metal–ligand coordination) are accountable for the self-aggregation of free porphyrin or metalloporphyrin compounds [41,42,43,44,45]. Unlike inorganic nanostructures, self-assembled porphyrin nanostructures are formed using several methods, including ionic self-assembly [46,47], reprecipitation [48,49], metal–ligand coordination [50,51], sonication-assisted self-assembly [52,53], and surfactant-assisted methods [54,55]. The morphologies of these organic nanostructures are not always uniform and are not suitable for numerous practical applications. Therefore, fabricating porphyrin-based nanoaggregates with specific dimensions is difficult. The fabrication of porphyrin-based nanostructures, compared to monomeric porphyrin units, not only changes the structural framework but also creates high structural modifications, including high thermodynamic stability, high porous surface area, interesting morphology, and outstanding photocatalytic degradation efficiency against pollutants under visible-light irradiation in an aqueous solution. The efficiency of harvesting solar energy in the visible region and the effective electron transfer properties of these materials make them unique compared to inorganic metal oxide nanoparticles. Therefore, the low cost, ease of synthesis, high efficiency, and high reproducibility of these nanomaterials make them outstanding candidates as visible-light photocatalysts.

In this review, we focus on the ongoing developments in this field, as well as the fabrication of distinct self-assembled porphyrin nano- and micro-structures with precise dimensions, and we highlight their photodegradation of pollutants from wastewater. 

## 2. Fabrication of Porphyrin-Based Nanostructures

The self-assembly property of porphyrin nanostructures is based on several interactions at the molecular level, such as π-π interactions, electrostatic interactions, coordination bonding interactions, and hydrogen bonding interactions. In the case of porphyrin-based nanostructures, particular molecular interactions among the inherent constituents directly affect the conditions for self-assembly and the successive stages of self-aggregation. Based on these interaction forces, various fabrication processes have been effectively used to synthesize porphyrin-based nanoaggregates via self-assembly, including ionic self-assembly, coordination-based aggregation, and reprecipitation with and without the help of surfactants. Porphyrin-based nanostructures with various morphologies obtained using different self-assembly approaches are discussed in the following sections. 

### 2.1. Reprecipitation Technique

**Figure 1:**
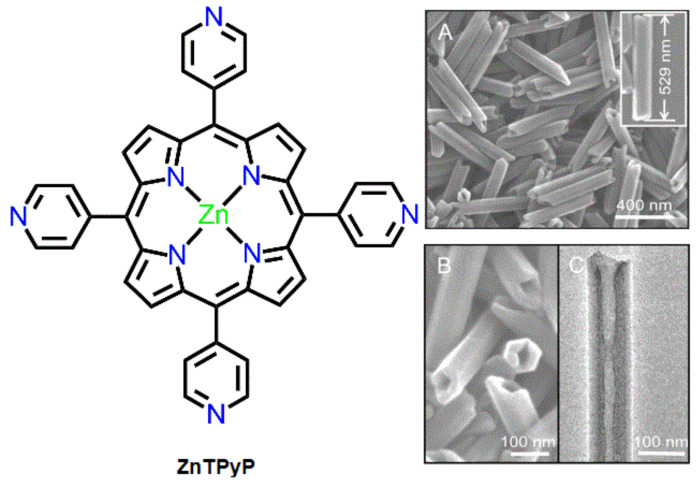
Morphology of ZnTPyP: SEM (**A**,**B**) and TEM (**C**). Reproduced from Ref. [56].

Reprecipitation is the easiest method for fabricating isolated porphyrin-based nanomaterials. Porphyrin nanoaggregates are fabricated via inoculation using this method. In this process, a highly concentrated solution of porphyrin dissolved in a non-polar solvent is injected into a polar solvent with vigorous stirring. This technique is referred to as the solvent exchange method. In some cases, surfactants are used to prevent agglomeration and control the structure of assembled nanostructures. Hollow-shaped hexagonal nanoprisms comprising ZnTPyP with a uniform size and shape were fabricated using reprecipitation methods [56]. The nanostructures were fabricated by mixing a ZnTPyP solution (dissolved in DMF) with CTAB (in water) at room temperature under vigorous stirring. Scanning electron microscopy (SEM) and high-resolution TEM (HRTEM) were used to determine the surface fabric of the nanoaggregates (Figure 1). The average length of these nanoprisms ranged from 517 to 541 nm, and their width ranged from 92 to 98 nm. The fence of the nanoprisms was approximately 30 nm thick. The aspect ratio and dimensions of these nanoprisms could be controlled by regulating the chemical proportion of ZnTPyP to CTAB. The absorption spectra of the nanoprisms showed a reduction in the monomeric band at 424 nm and the *J*-aggregate band at 460 nm. XRD data confirmed that the axial coordination between the pyridyl-N atom and Zn^II^ metal was responsible for this type of self-assembly. Interestingly, 1D hexagonal hollow nanoprisms can easily self-aggregate into uniform 3D architectures when the solvent evaporates. 

**Figure 2:**
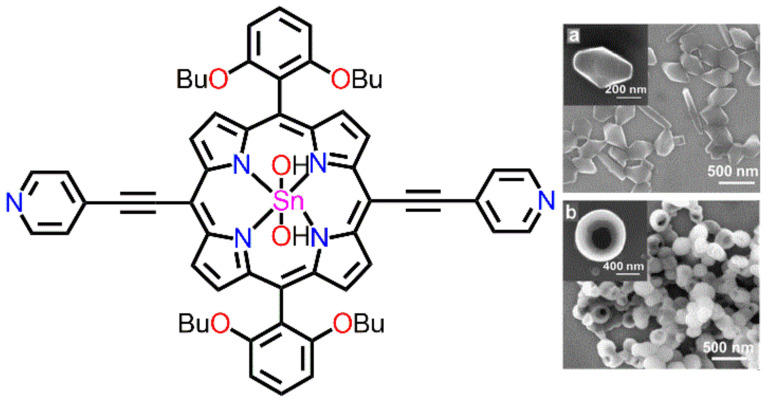
SEM image of tin dipyridylporphyrin under various conditions: room temperature (**a**) and 90 °C (**b**). Adapted from Ref. [57].

Nguyen et al. described the use of a reprecipitation method without surfactants to construct porphyrin nanostructures from tin(IV)dipyridylporphyrin (Figure 2) [57]. An ethanolic solution of tin(IV)dipyridylporphyrin was added to water at room temperature. After several days, approximately 280 nm long oval-shaped flat crystals were obtained from this heterogeneous mixture (Figure 2a). The temperature-dependent morphology of the porphyrins was also investigated. Amorphous hollow spheres (diameter approximately ~350 nm) were obtained after injecting an ethanolic solution of tin(IV)dipyridylporphyrin into water at 90 °C (Figure 2b). This study describes a new method for tuning the extension of molecular nanocrystals in solutions under dynamic flourishing conditions, which can be assembled into substantial structures using CTAB.

**Figure 3:**
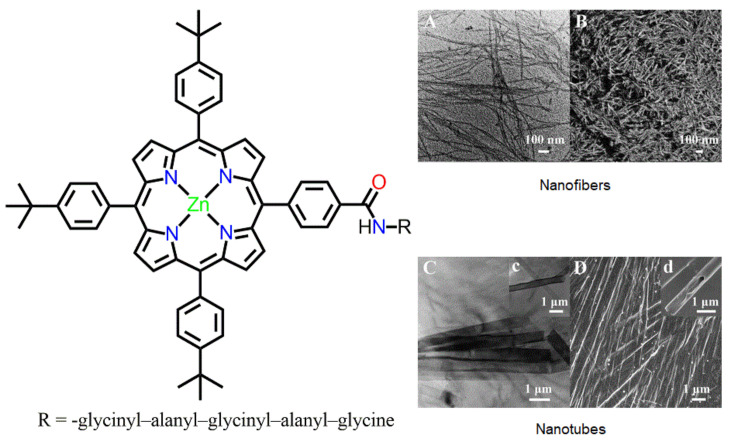
Self-assembled nanostructures derived from *n*-hexane and THF (3:1): HRTEM (**A**) and SEM (**B**) of nanofibers. Nanostructures fabricated from THF and water (1:3): HRTEM (**C**) including an enlargement (**c**) and SEM (**D**) including an enlargement (**d**) of nanotubes. Adapted from Ref. [58].

Optically active covalently linked porphyrin–pentapeptide chains show a solvent-dependent morphology (Figure 3) [58]. Nanofibers were fabricated from THF (tetrahydrofuran) and *n*-hexane (1:3) mixtures, and nanotubes were obtained from THF and water (1:3) mixtures. In the case of the nanotubes, XRD results verified that a dimeric bilayer conformation was formed between the two conjugate molecules via intermolecular hydrogen bonding. However, a *right*-handed helix of porphyrin moieties was observed in the case of the nanofibers. The fabricated nanostructures exhibited good semiconductor properties. 

**Figure 4:**
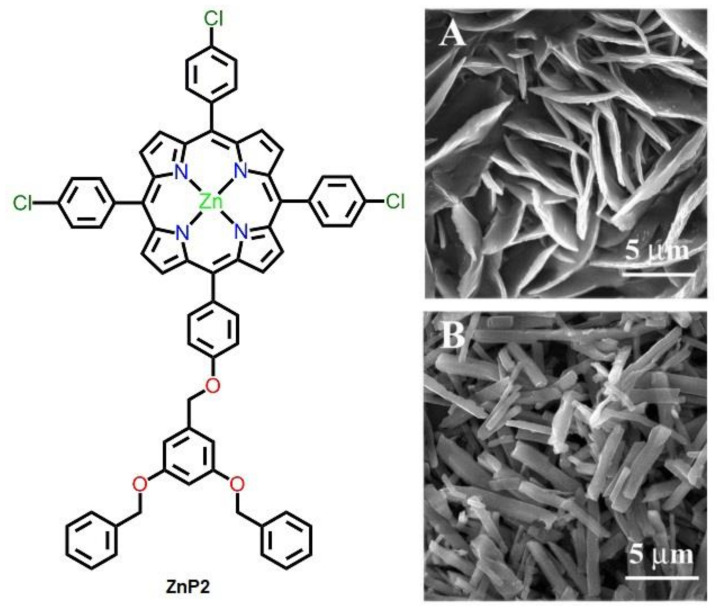
Solvent-induced SEM image of ZnP2: *n*-hexane (**A**) and methanol (**B**). Adapted from Ref. [59].

Occasionally, solvents can control the supramolecular formation of porphyrin-based nanostructures. Zhu et al. reported that a zinc complex of benzyloxy-based porphyrin (ZnP2) displayed micrometer-sized leaves in a non-polar solvent such as *n*-hexane, whereas twisted nanorods were observed in methanol (Figure 4) [59]. This observation can be attributed to the different kinds of intermolecular interactions (π-π stacking interactions and hydrogen bonding interactions) arising between ZnP2 molecules in the presence of either polar or non-polar solvents. The width of the micrometer-shaped leaves was approximately 50 nm. The length of the twisted nanorods varied from 4 to 8 μm, while the width ranged from 500 to 800 nm, and the height ranged from 500 to 700 nm. 

**Figure 5:**
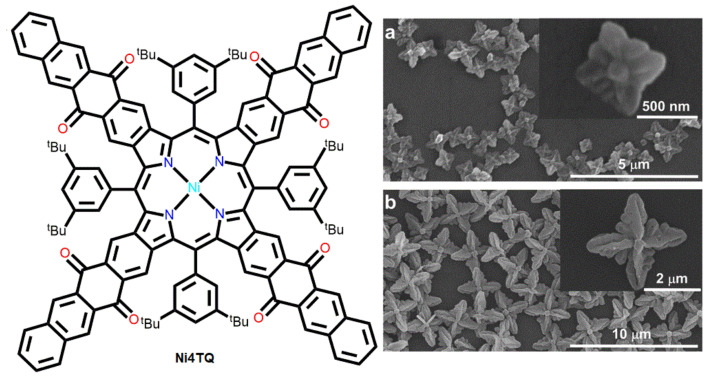
Solvent-regulated SEM image of Ni4TQ microstructures acquired in (**a**) MeOH: toluene (9:1) and (**b**) MeOH: toluene (8.5:1.5). Adapted from Ref. [60].

The ratio of “good solvent” and “bad solvent” can control the morphology of porphyrin nanostructures. Tetracenequinone units fused to nickel porphyrin (Ni4TQ) showed uniform flower-shaped biomorphs in mixed-solvent systems (MeOH and toluene) (Figure 5) [60]. Ni4TQ displayed regular four-leaf clover-like structures via drop casting in a mixed-solvent system (MeOH:toluene = 9:1). The diagonal length of this square clover was approximately 500 nm (Figure 5a). However, larger (~2.5 μm) flower-shaped microstructures were observed (MeOH:toluene = 8.5:1.5) as the MeOH percentage was increased (Figure 5b). 

**Figure 6:**
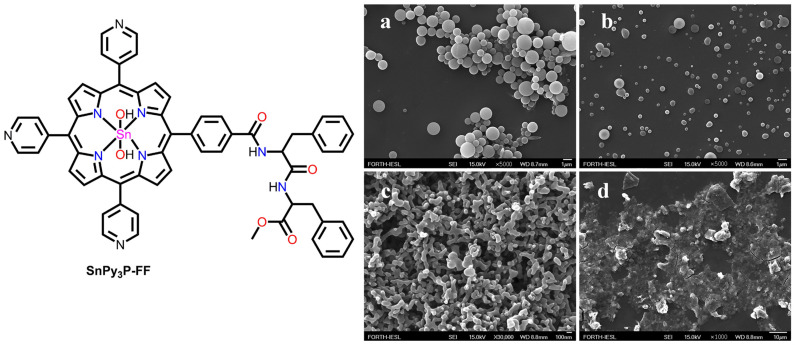
SEM images of nanostructures derived from different solvents: (**a**) DCM/MeOH (2:8), (**b**) MeOH/HFIP (8:2), (**c**) heptane/DCM (8:2), and (**d**) MeOH/THF (8:2). Adapted from Ref. [61].

Coutsolelos et al. reported the solvent-dependent morphologies of tin(IV)porphyrin-based peptide conjugates (SnPy_3_P-FF) [61]. The precursor tripyridyl porphyrin macrocycles were covalently linked to the diphenylalanine dipeptide. SnPy_3_P-FF exhibited a solvent-dependent self-assembled morphology. In a typical procedure, SnPy_3_P-FF was dissolved in a “good solvent” (DCM, THF, or HFIP (1,1,1,3,3,3-hexafluoroisopropanol)), and then a “bad solvent” (MeOH or heptane) was injected to induce the self-assembly process. It exhibited spherical nanostructures in mixed-solvent systems, such as DCM/MeOH (2:8). Amorphous aggregates were obtained from THF, and agglomerated spheres were obtained from heptane/DCM (8:2) (Figure 6).

### 2.2. Ionic Self-Assembly (ISA)

**Figure 7:**
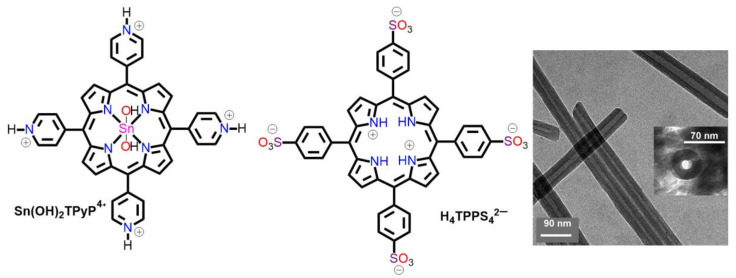
TEM images of ionic self-assembled nanostructures fabricated from cationic Sn(OH)_2_TPyP^4+^ and anionic H_4_TPPS_4_^2−^. Adapted from Ref. [62].

ISA is a common method for the construction of nano- and micro-structured compounds. It involves the integration of various oppositely charged species, and they are held by electrostatic interactions. In binary porphyrin systems, anionic and cationic porphyrins promote self-assembly via electrostatic interactions between the oppositely charged molecules. So far, in porphyrin binary systems, the di-acid form of H_4_TPPS_4_^2−^ has been widely used. Shelnutt et al. reported porphyrin nanotubes from the combination of H_4_TPPS_4_^2−^ with cationic Sn(OH)_2_TPyP^4+^ [62]. High-resolution TEM images of the porphyrin nanotubes (Figure 7) revealed that their lengths varied from a few nanometers to a few micrometers, with their diameters varying from 50 to 70 nm. The wall thickness of the nanotubes was approximately 20 nm. It was confirmed that the porphyrin molecules were stacked in the *J*-aggregate mode, which resulted in the generation of red-shifted UV–visible absorption bands at 714 and 496 nm from the complementary bands of the starting porphyrin monomer (Figure 7).

**Figure 8:**
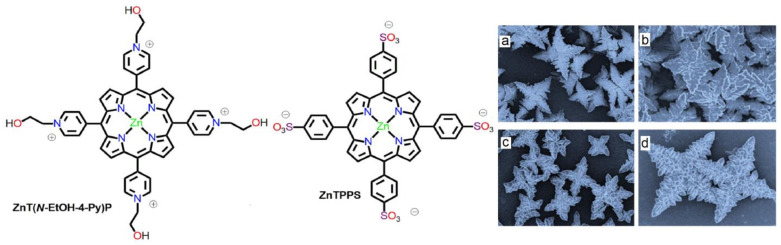
Morphology of ionic self-assembled nanomaterials constructed from cationic ZnT(*N*-EtOH-4-Py)P and anionic ZnTPPS at several temperatures. (**a**) 10 °C, (**b**) 23 °C, (**c**) 60 °C, (**d**) 80 °C. Adapted from Ref. [63].

Shelnutt et al. reported biomorph-like nanostructures with the configuration of a four-leaf clover [63]. For this, they used equal proportions of anionic ZnTPPS and cationic ZnT(*N*-EtOH-4-Py)P. Using SEM, the average diameter of these clovers was found to be roughly 5 ± 2 mm, and the shapes varied with temperature and other conditions (Figure 8). These self-assembled nanostructures were sufficiently stable for several months and could be utilized for effective hydrogen generation. Porphyrin clovers exhibit higher photocatalytic H_2_ evolution activity than individual porphyrins. This is due to the fact that, unlike monomeric porphyrins, the binary solids in clovers engage in cooperative behavior.

**Figure 9:**
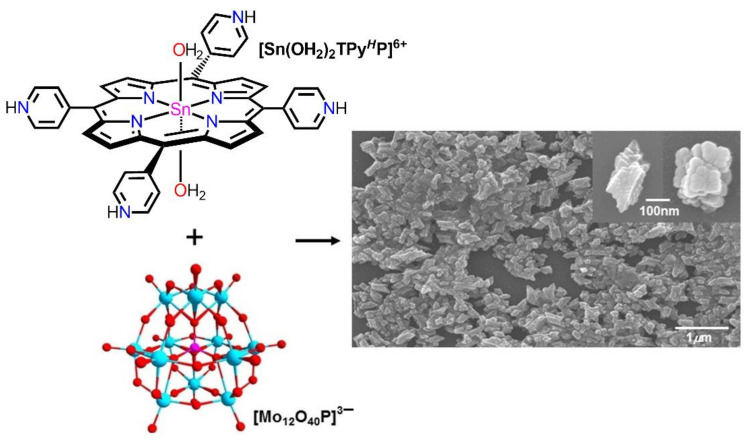
Morphology (SEM) of nanostructures fabricated from cationic tin(IV)porphyrin and anionic polyoxomolybdate. Adapted from Ref. [64].

Sometimes, ionic self-assembly is possible by combining charged porphyrin building blocks with oppositely charged nonporphyrinic species. In 2015, Kim et al. reported porphyrin–polyoxometalate-based heterostructures using electrostatic interactions between polyoxomolybdate anions and tin(IV)porphyrin cations (Figure 9) [64]. DLS calculations confirmed that the diameter of these nanoparticles in H_2_O was approximately 245 nm with a slender disposition in size. These nanomaterials were exceptionally stable in both neutral and acidic media. Moreover, ionic self-assembled nanomaterials have been used as photosensitizers for H_2_ generation under sunlight. 

**Figure 10:**
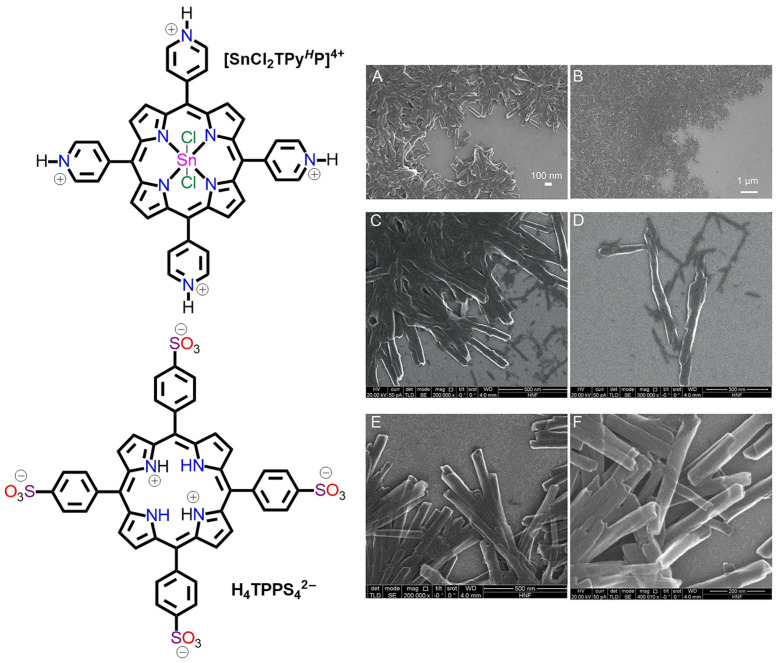
Morphology of fabricated nanoaggregates obtained from the combination of [SnCl_2_TPy*^H^*P]^4+^ with H_4_TPPS_4_^2−^ in solution at pH = 2.0. Concentration ratio of [SnCl_2_TPy*^H^*P]^4+^: H_4_TPPS_4_^2−^; 1:1 (**A**,**B**), 1:5 (**C**,**D**), and 5:1 (**E**,**F**). Adapted from Ref. [65].

Mourzina et al. prepared various porphyrin-based nanostructures by varying the ratio of cationic porphyrin [SnCl_2_TPy*^H^*P]^4+^ and anionic porphyrin H_4_TPPS_4_^2−^ in solutions (Figure 10) [65]. SEM images showed that these hollow tubular nanostructures had a length of 0.4–0.8 μm with open ends. The inner and outer diameters were 7–15 nm and 30–70 nm, respectively. These nanotube frameworks demonstrated a photoconductivity of (3.1 ± 0.9) × 10^−4^ s m^−1^ under visible light and behaved as a metal-like material. 

**Figure 11:**
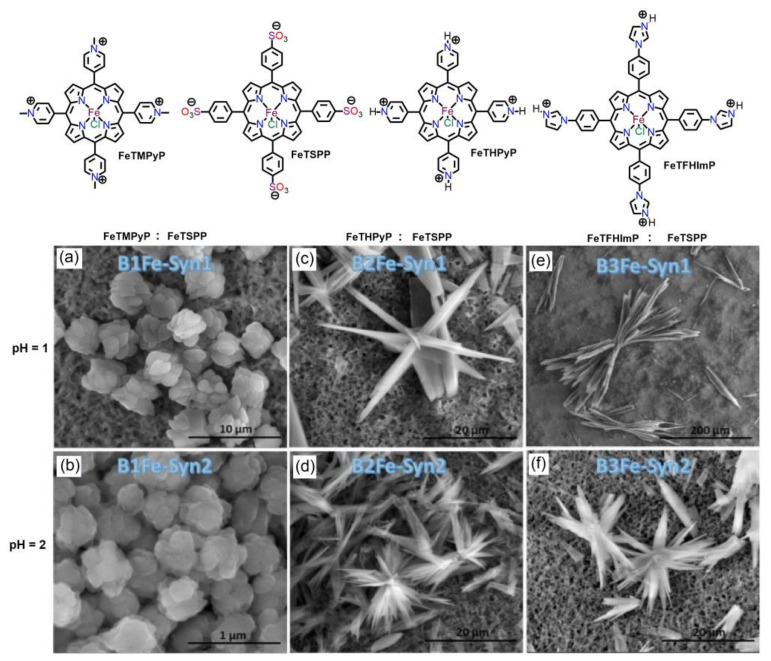
SEM images of ionic self-assembled nanostructures constructed from the combination of anionic FeTSPP with various cationic iron porphyrins in solution: (**a**,**c**,**e**) at pH = 1. (**b**,**d**,**f**) at pH = 2. Adapted from Ref. [66].

Medforth et al. reported various nanostructures using the ionic self-assembly of anionic Fe(III)porphyrin (FeTSPP) with several cationic Fe(III)porphyrin building blocks in aqueous media [66]. The shape of the aggregates depended on the substituents on the cationic porphyrin and the pH of the solution (Figure 11). Hyperbranched structures were obtained by combining anionic FeTSPP with protonated pyridinium porphyrin (FeTHPyP) or protonated imidazolium porphyrin (FeTFHImP). In contrast, globular assemblies of sheets were obtained using N-methylpyridinium porphyrin (FeTMPyP). These fabricated nanoaggregates showed higher catalase activity than individual porphyrin molecules.

### 2.3. Surfactant-Assisted Self-Assembly (SASA)

**Figure 12:**
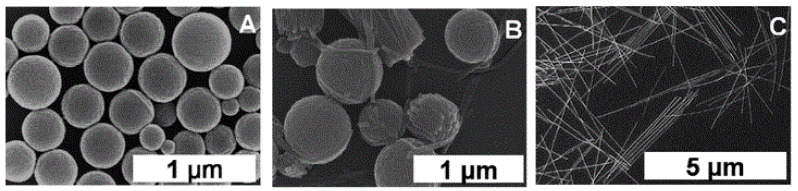
SEM images of the formed porphyrin nanoarchitectures from ZnTPyP with different aging times: (**A**) nanospheres (t = 0), (**B**) heterostructures (t = 5 h), (**C**) nanofibers (t = 10 days). Adapted from Ref. [67].

Thus far, most porphyrin nanostructures are organized in aqueous media and depend on the pH, temperature, and concentration. However, surfactants act as a template in water and can influence the self-organization of nanoaggregates for practical applications. In 2012, Liu et al. reported on self-organized 1D nanofibers obtained using a surfactant-assisted reprecipitation method [67]. In a typical process, ZnTPyP was dissolved in CHCl_3_, and CTAB was dissolved in water. The ZnTPyP solution was then slowly injected into the CTAB solution with vigorous stirring. The diameters of the fabricated spherical nanospheres ranged from 0.1 to 1 μm. These self-assembled nanospheres were gradually converted into 1D nanofibers via aging under the appropriate conditions (Figure 12). The average length was found to be approximately 4.5 μm, and the diameter was approximately 50 nm. This result provides a novel approach for the creation of tractable porphyrin-based 1D nanoaggregates using surfactant-assisted self-assembly.

**Figure 13:**
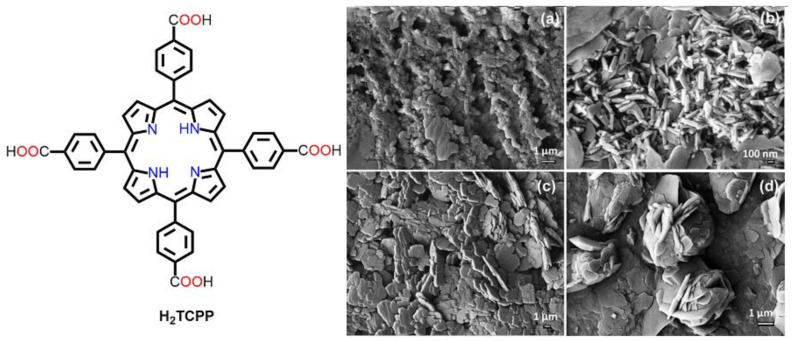
SEM images of various morphologies as a function of different stirring times. (**a**) 15 min, (**b**) 60 min, (**c**) 6 h, (**d**) 48 h. Adapted from Ref. [68].

Patra et al. synthesized different surfactant-assisted assembled nanostructures of H_2_TCPP by varying the stirring time [68]. In a typical reaction, H_2_TCPP was dissolved in a dilute NaOH solution. CTAB was dissolved in a dilute HCl solution in another container. The alkaline solution of H_2_TCPP was then mixed with the acidic CTAB solution under persistent stirring (~1200 rpm), and the morphologies were examined after 15 min, 60 min, 6 h, and 48 h (Figure 13). Spherical nanoaggregates with a diameter of 100 nm were observed after 15 min of stirring (Figure 13a). These nanospheres were converted into rod-shaped nanoaggregates after stirring for 1 h (Figure 13b). The average length was approximately 230 nm, with a width of 73 nm. These rod-shaped aggregates were converted into flake-shaped microaggregates after stirring for 6 h (Figure 13c). The average length of the flakes was roughly 3.5 μm, and the width varied from 400 to 500 nm. At the end, flower-shaped nanoaggregates (Figure 13d) were assembled, with a size of approximately 4.5 μm after 48 h of stirring. The authors also reported that, without the surfactant CTAB, these nanoarchitectures from H_2_TCPP would not have been achieved.

**Figure 14:**
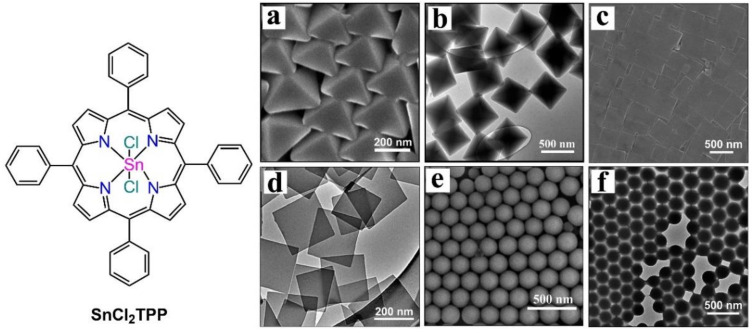
Different surfactant-assisted morphologies as a function of surfactant. SEM (**a**) and TEM (**b**) morphologies of octahedral arrays from CTAB. SEM (**c**) and TEM (**d**) morphologies of nanosheets from CATB and emulsion. SEM (**e**) and TEM (**f**) morphologies of nanospheres from SDS. Adapted from Ref. [69].

In some cases, the morphology of nanostructures differs depending on the surfactant used for self-assembly. Bai et al. observed that surfactants were crucial for adjusting the morphology and arrangement of porphyrin molecules. They reported two different morphologies when they changed the surfactant from CTAB to SDS (sodium dodecyl sulphate) [69]. In a typical process, SnCl_2_TPP was dissolved in chloroform, and the surfactants (CTAB or SDS) were dissolved in water. An emulsion was formed by vigorously mixing the two solutions. After that, the solvent was vaporized from the heterogeneous system by heating at 60 °C. Octahedral arrays were obtained from the CTAB solution, nanosheets were obtained from the CTAB and emulsion, and microspheres were obtained from SDS. For a regular octahedron, the average length was estimated to range from 320 to 380 nm. However, the average length of the square-shaped nanosheets was calculated to be 400 ± 100 nm. The average diameter of the microspheres varied from 200 nm to 220 nm (Figure 14). 

**Figure 15:**
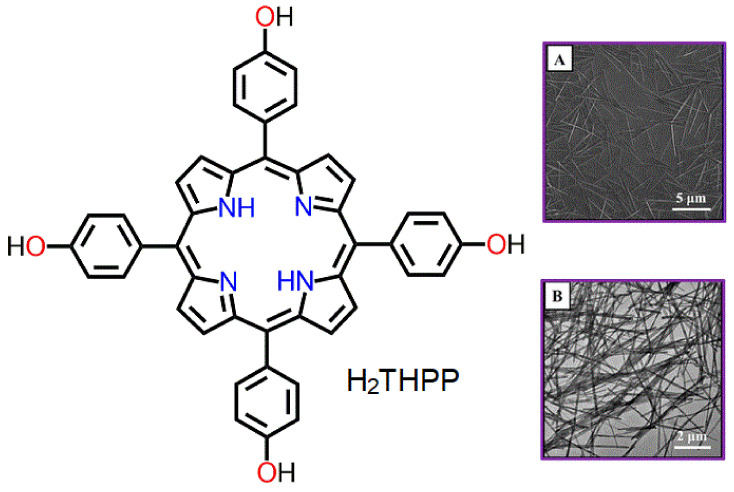
Surfactant-assisted nanowire morphology of H_2_THPP. SEM (**A**) and TEM (**B**). Adapted from Ref. [70].

Fan et al. reported one-dimensional (1D) porphyrin nanomaterials using SASA [70]. The nanomaterials were constructed using H_2_THPP as the starting material. In a typical process, an acidic (pH 2.70) CTAB (2.5 mM) solution was added dropwise to an alkaline solution of H_2_THPP (0.0 mM) under vigorous stirring for 48 h. The fabricated nanowires were regular in shape. The average length of these monodispersed nanowires was approximately 4.25 μm, with a width of 110 nm (Figure 15). The 1D self-assembled nanowires of H_2_THPP were found to be almost 20 times better for hydrogen generation than monomeric H_2_THPP powder. This is due to the strong delocalization of excited-state electrons in the nanowire morphology, which improves the lifetime of photogenerated electron–hole pairs and enhances hydrogen production.

**Figure 16:**
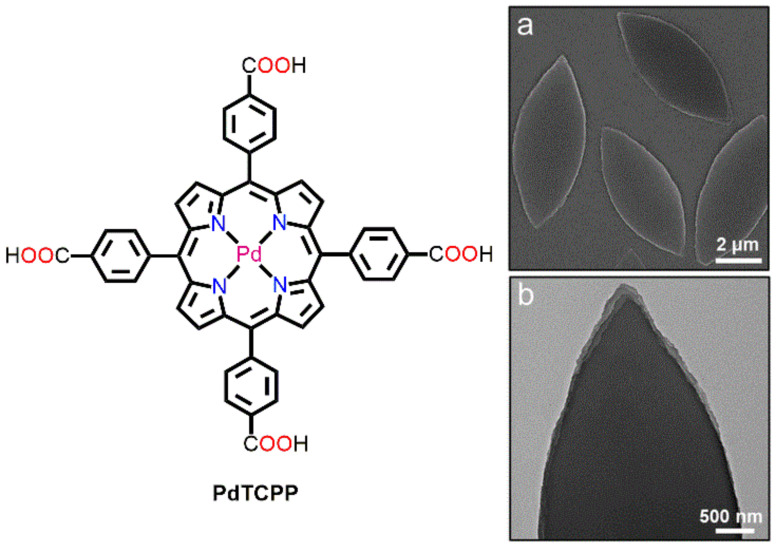
Surfactant-assisted PdTCPP nanoleaves from DASS (15 mM) and porphyrin (1 mM). Morphology: (**a**) SEM and (**b**) TEM. Adapted from Ref. [71].

Zhu et al. reported porphyrin-based nanoleaves using the surfactant-assisted method, with the Pd^II^ complex of H_2_TCPP (PdTCPP) as the starting molecule and DASS as the surfactant [71]. Using a typical process, a DMF solution of PdTCPP was injected into an aqueous DASS solution and vigorously stirred at room temperature. Nanoleaves were collected by centrifuging the above solution. The nanoleaves were regular in shape and had an average size of 1.64 μm × 5.43 μm, with a thickness of approximately 200 nm (Figure 16).

### 2.4. Metal–Ligand Coordination-Assisted Self-Assembly

**Figure 17:**
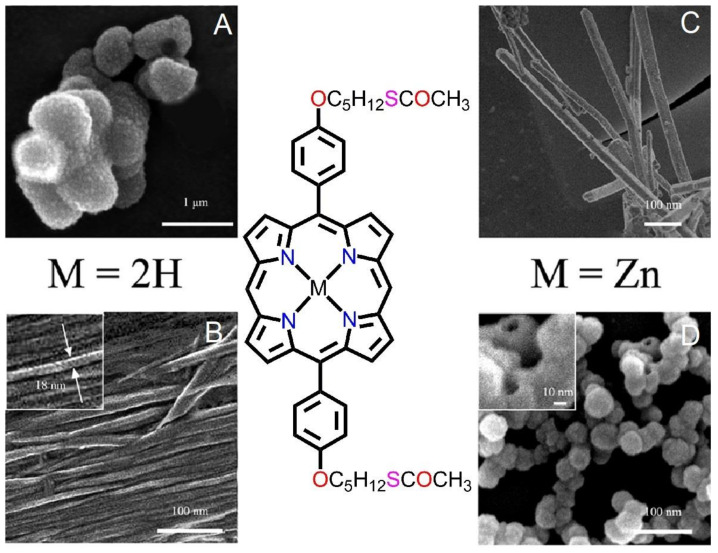
SEM images of porphyrin nanostructures: (**A**,**C**) in methanol, (**B**,**D**) in *n*-hexane. Zoom-in image of (**B**,**D**). Adapted from Ref. [72].

In the case of metalloporphyrins, the metal–ligand coordination bond plays a pivotal role in the conformation of molecular packing and controls the conformation of self-assembled nanomaterials. Jiang et al. explored the self-assembly of H_2_[DP(CH_3_COSC_5_H_10_O)_2_P] and its Zn complexes [72]. Both compounds were dissolved in two solvents (methanol and *n*-hexane) and then dropped onto a carbon-coated gauze for an SEM analysis. Metal-free porphyrin (M = 2H) formed nanoscale hollow spheres (diameter of ~700 nm) in methanol (Figure 17A) and nanoribbons (average width of ~18 nm) in *n*-hexane (Figure 17B). In contrast, the zinc complex (M = Zn) formed nanorods (50 nm in width and 800 nm in length) in methanol (Figure 17C) and nanospheres (diameter of ~40 nm) in *n*-hexane (Figure 17D). In these observations, it was evident that the fabrication of these nanostructures depended primarily on two factors: intermolecular metal–ligand interactions (O-Zn coordination) and π-π stacking interactions between tetra-pyrrole rings. However, for the metal-free porphyrin compound (M = 2H), π-π interactions controlled the fabrication of nanoaggregates. In contrast, metal–ligand interactions were responsible for the different nanostructures in the zinc complex (M = Zn).

**Figure 18:**
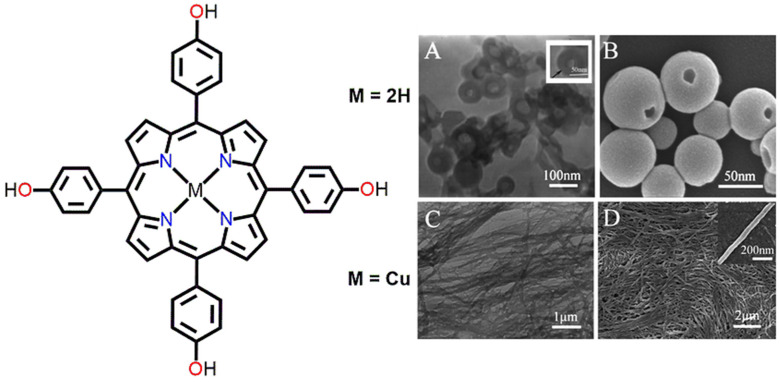
SEM images of porphyrin nanostructures derived from water; TEM (**A**,**C**) and SEM (**B**,**D**). Zoom-in image of (**A**,**D**). Adapted from Ref. [73].

Zhang et al. reported porphyrin nanoaggregates of H_2_THPP and its copper(II) complex CuTHPP in an aqueous solution [73]. Hollow nanospheres were obtained from H_2_THPP. The average particle diameter was approximately 70 nm. However, one-dimensional (1D) nanoribbons with a 10 µm length and a 70 nm width were obtained from CuTHPP (Figure 18). In the case of H_2_THPP, intermolecular π-π interactions in cooperation with hydrogen bonding interactions were responsible for the hollow-shaped nanospheres. In contrast, in the case of CuTHPP, intermolecular Cu–O metal–ligand coordination bonding was responsible for the ribbon-like morphology.

**Figure 19:**
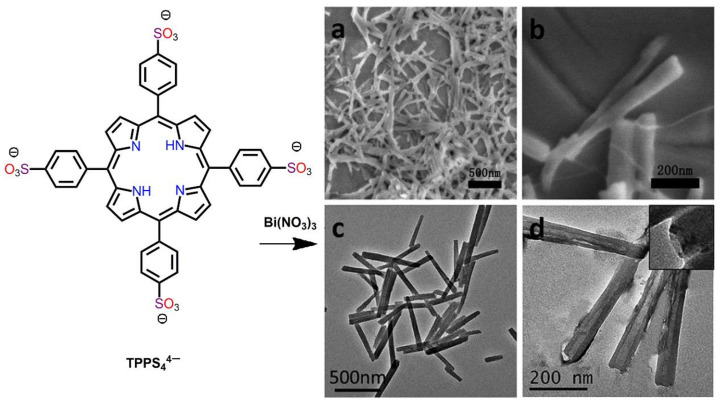
SEM images of porphyrin nanostructures derived from TPPS_4_^4−^ with the addition of Bi(NO_3_)_3_ in water (pH = 3.6). SEM; low (**a**) and high (**b**). TEM; low (**c**) and high (**d**). [Bi^3+^] = 0.2 mM, [TPPS_4_^4−^] = 0.1 mM. Adapted from Ref. [74].

Yan et al. reported TPPS_4_-Bi nanotubes fabricated via out-of-plane coordination assisted by bismuth porphyrins [74]. These nanotubes were prepared from the reaction of meso-tetra(sulfonatophenyl)porphine (TPPS_4_^4−^) with Bi(NO_3_)_3_ in water. The lengths, widths, and thickness of these nanotubes were approximately 500 ± 100 nm, 50 ± 5 nm, and 15 ± 5 nm, respectively (Figure 19). The Bi^3+^ metal ions placed between two TPPS_4_^4−^ units at a dislocation angle force a staggered arrangement of TPPS_4_^4−^. This not only prohibited π-π stacking overlap but also permitted Bi-TPPS_4_ *J*-aggregation to conquer the self-quenching sequel.

**Figure 20:**
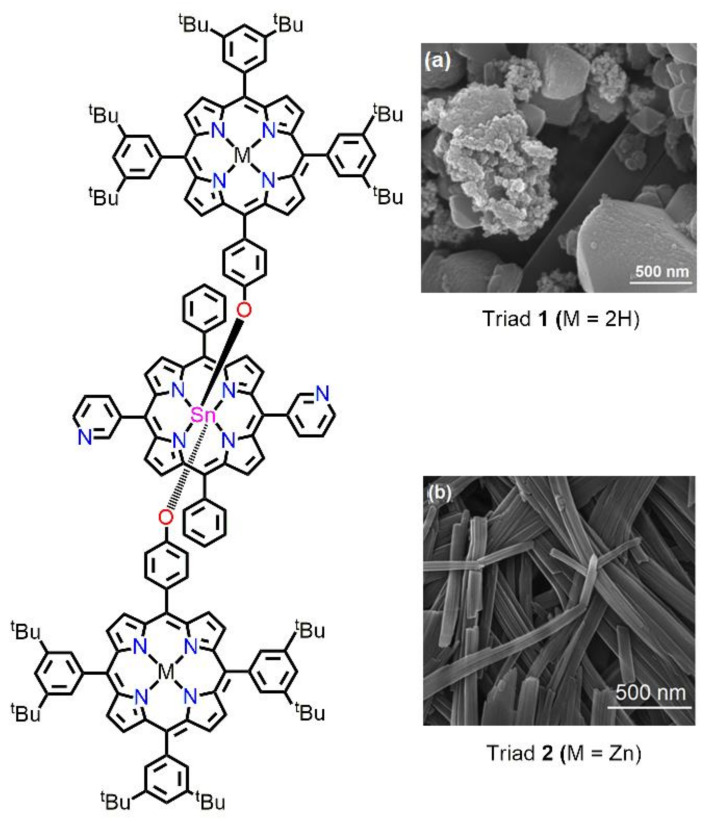
SEM images of porphyrin nanostructures derived from the drop casting of a toluene solution of porphyrin triad molecules onto copper tapes. M = 2H, (**a**); M = Zn, (**b**). Adapted from Ref. [75].

Recently, Kim et al. fabricated porphyrin-based nanostructures from porphyrin triads via metal–ligand coordination self-assembly [75]. These triads were constructed by reacting Sn(IV)porphyrin with various porphyrin derivatives. The robust disposition of Sn(IV)porphyrin for the coordination of aryloxides and carboxylates was the main reason for the formation of steady coordination arrays. In triad **2**, effective cooperative coordination among the 3-pyridyl N atoms of tin(IV)porphyrin with the Zn atoms of axial zinc porphyrins followed by π-π interactions led to the fabrication of regular nanofibers with an average width varying from 10 to 22 nm. The lengths of these nanofibers varied from nanometers to micrometers. It was interesting that the absence of Zn(II) metal in triad **1** resulted in non-uniform nanostructures, as only π-π stacking associations were dominant for aggregation. Notably, in the presence of an external ligand (pyrrolidine), the aggregation of the nanofibers was disrupted (Figure 20). 

**Figure 21:**
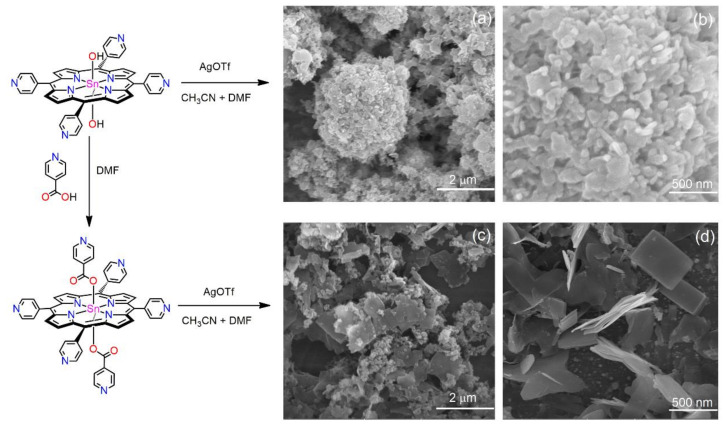
SEM images of porphyrin nanostructures derived from the addition of AgOTf to a solution of Sn(IV)porphyrin precursors. Without axial ligation (**a**,**b**), with axial ligation (**c**,**d**). Adapted from Ref. [76].

In another study, Kim et al. constructed porphyrin-based nanostructures based on metal–ligand coordination self-assembly [76]. The addition of silver salt (AgOTf) to a solution of the two tin(IV)porphyrin precursors led to the formation of different nanoaggregates. The axial ligation of tin(IV)porphyrin centers not only transformed the conformations of the supramolecular arrays but also generated high structural configurations, including a high porosity and chemical stability and an interesting morphology. Without axial ligation, the fused nanoparticles hooked on to each other, with an average length varying from 200 nm to 400 nm. With axial ligation, a rectangular flake-shaped nanoaggregate formed. The average width of these nanoflakes was approximately 25 nm, with the average length ranging from 250 to 600 nm (Figure 21).

### 2.5. Miscellaneous Processes

**Figure 22:**
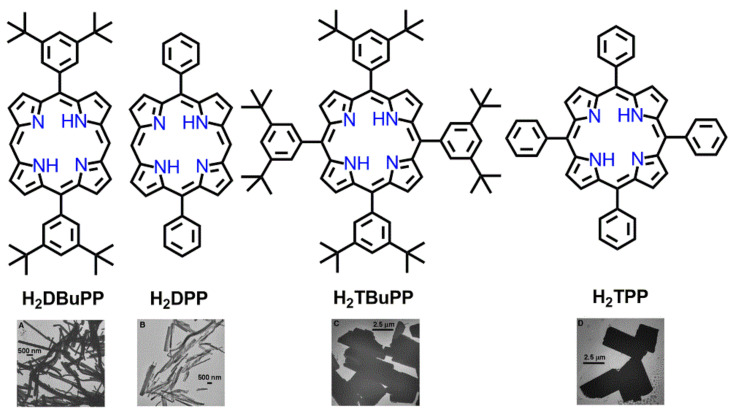
SEM images of sonication-assisted porphyrin nanostructures. Adapted from Ref. [52].

Porphyrin-containing nanoaggregates can also be constructed using other unfamiliar techniques. Hasobe et al. fabricated supramolecular porphyrin-based nanoaggregates via sonication [52]. A toluene solution of the porphyrin was mixed nine times with acetonitrile (*v*/*v*), and the solution was sonicated for half an hour at 288 K to fabricate porphyrin-based macroscopic materials (Figure 22). No nanostructures formed in the absence of sonication.

**Figure 23:**
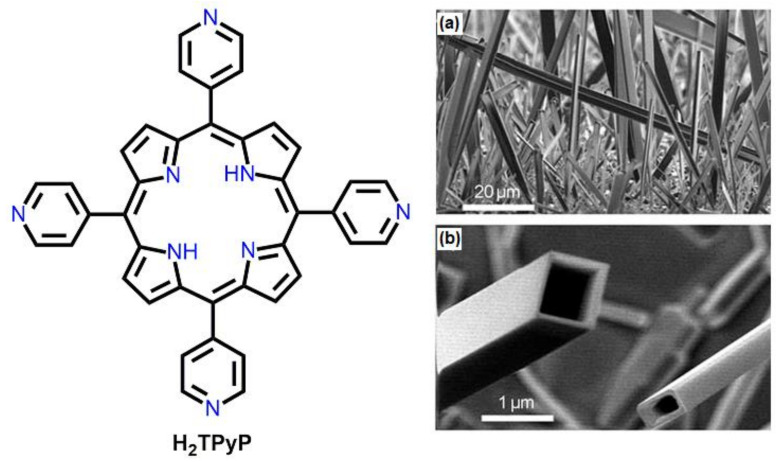
SEM images of nanotubes of H_2_TPyP prepared using a vaporization–condensation–recrystallization method. Cross-section (**a**), high resolution (**b**). Adapted from Ref. [77].

Choi et al. prepared 1D porphyrin-based nanotubes using a vaporization–condensation–recrystallization method [77]. In this process, H_2_TPyP solid powder was heated to 450 °C on a carbon-coated Si(100) substance in an inert gas atmosphere. The rectangular nanotubes were organized in the end domain of the furnace temperature (~350 °C). Crystallographic data confirmed that the abovementioned nanotubes formed by the self-stacking of H_2_TPyP building blocks via π-π, H-π, and hydrogen bonding interactions (Figure 23). 

**Figure 24:**
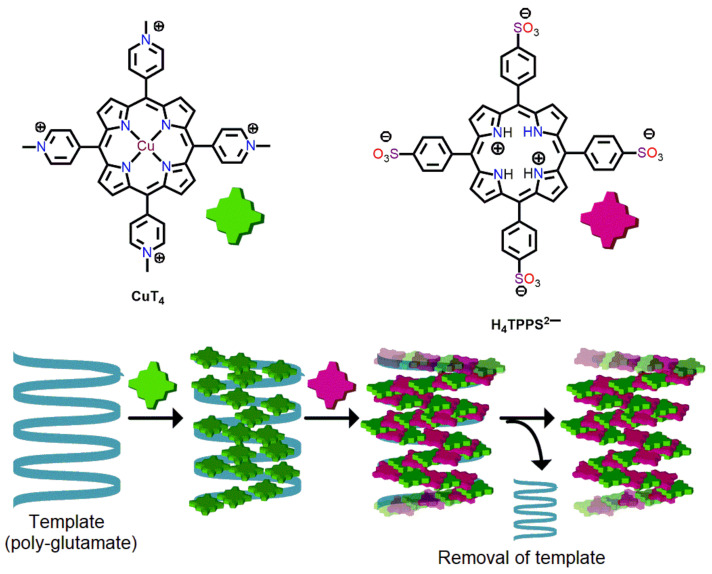
Template-assisted self-assembly of porphyrin nanoaggregates. Adapted from Ref. [78].

Purello et al. demonstrated a rare self-aggregation technique for the fabrication of porphyrin nanoaggregates [78]. In this method, a tetra-cationic porphyrin (CuT4) was assembled on a poly-glutamate template. Afterwards, a tetra-anionic porphyrin (H_4_TPPS^2−^) was assembled in the pre-templated aggregates. Self-assembled porphyrin nanoaggregates were obtained by removing the template (Figure 24). 

**Figure 25:**
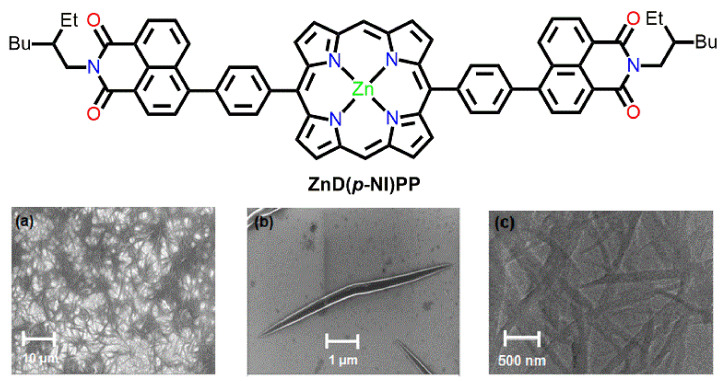
The morphology of the nanowire is derived from ZnD(*p*-NI)PP. SEM (**a**,**b**) and TEM (**c**) Adapted from Ref. [79].

Zhu et al. prepared porphyrin-based nanowires from naphthalimide-based linear metalloporphyrin ZnD(*p*-NI)PP using a drop-casting method [79]. Porphyrin nanowire films were fabricated by dropping a CHCl_3_ solution onto a silica substrate and copper grids and then drying them in air. The widths of these nanowires varied from 100 nm to 250 nm, with an average length ranging from 1.5 to 5.0 μm. In this case, ZnD(*p*-NI)PP was easily aggregated into nanowires with a distinct fabric via the cooperative π-π stacking interactions between the naphthalimide and porphyrin frameworks (Figure 25). 

## 3. Application of Porphyrin-Based Nanostructures for Photocatalytic Remediation of Wastewater

In a photocatalytic reaction, photocatalysts are triggered by the absorption of adequate photon energy (*hv*) (≥bandgap energy (E_g_)) [80]. This process will create a charge separation due to the boosting of *e*^−^ from the lowest VB energy level to the highest CB energy level, thus creating a pair of holes. A photocatalyzed reaction can be favored if the recombination of hole pairs is prevented. This is because the activated electrons react with dye to generate reduced species, and the generated holes react with dye to produce an oxidized product. Alternatively, the photogenerated electrons may react with dissolved O_2_ and generate superoxide radical anions (O_2_^−•^). Furthermore, photogenerated holes may react with H_2_O or OH^−^ and oxidize them into hydroxyl radicals (OH^•^). Sometimes, other oxidants such as peroxide radicals may be generated for photodecomposition. The resulting hydroxyl radicals and superoxide radical anions are strong oxidizing agents that can degrade toxic dyes to lower-molecular-weight compounds and finally mineralize them to CO_2_ and H_2_O. The appropriate reactions on the catalyst surface for the decay of toxic pollutants can be expressed as follows:

P + *hv*
→
P^*^[*h*^+^_(VB)_ + *e*
^−^_(CB)_] (1)

H_2_O + P^*^[*h*^+^_(VB)_] → P + OH^•^ + H^+^ (2)

OH^−^ + P^*^[ *h*^+^_(VB)_] → P + OH^•^ (3)

O_2_ + P^*^[*e*
^−^_(CB)_] → P + O_2_^−•^ (4)

O_2_^−•^ + H^+^ → HO_2_^•^ (5)

h_VB_^+^ + pollutant → oxidation products (6)

e_CB_^−^ + pollutant → reduction products (7)

Pollutant + OH^•^ → degraded products (8)

O_2_^−•^ + pollutant → degraded products (9)

Moreover, the photocatalytic activity of a photocatalyst bank depends on several important factors, such as the surface area, bandgap, recombination energy of hole pairs, surface morphology, active sites, crystallinity, phase composition, penetration of light through the photocatalysts, and adsorption capability of dyes on the surface of the photocatalysts. Owing to their vast morphologies and functional properties, porphyrin-based self-assembled nanomaterials have been extensively investigated for the separation of pollutant dyes from wastewater [75,76]. This is because porphyrin nanomaterials can be readily produced, and their morphology can be easily managed. Tuning the size and morphology of nanomaterials has a favorable effect on their properties, such as the light-harvesting properties, surface area, active sites and defect sites, movement of hole pairs along the morphology, and absorbance of dyes. These robust materials can directly harvest photon energy to produce radicals, prevent the degradation of energetic species, and increase their recyclability. Porphyrin-based nanostructures show a higher photocatalytic ability than monomeric amorphous porphyrins. This is because of the strong π-π interactions in *J*- or *H*-type interactions. Porphyrin nanostructures have excellent semiconductor properties and can improve charge separation during photocatalytic process. Generally, porphyrin-based nanomaterials absorb photon energy in the visible range, and electrons in the VB jump to the CB to generate electron–hole pairs. A strong electronic delocalization of these charged species occurs on the surface of porphyrin-based nanostructures. This delays the recombination of electron–hole pairs and thus enhances their photodegradation performance. Photogenerated electron–hole pairs participate in the degradation process to generate reactive oxygen species and oxidize pollutants to nontoxic CO_2_ and H_2_O. 

Each porphyrin-based nanostructure is different in terms of morphology. Each morphology has its own surface properties, such as surface energy, surface area, light-harvesting ability, and number of active and defect sites. This affects the delocalization of photogenerated charge species on the surface of the catalyst and alters the recombination energy of electron–hole pairs. Therefore, photocatalytic performance is controlled by the morphology of porphyrin-based nanostructures [75,76]. 

Reactive oxygen species generated during the photocatalytic process are evident in radical trapping experiments using scavengers or in electron spin resonance experiments, such as when *tert*-butanol (^t^BuOH) is employed to seize ^•^OH, NaN_3_ (sodium azide) is employed to seize ^1^O_2_, *p*-BQ (*para*-benzoquinone) is employed to seize O_2_^−•^, and Na_2_-EDTA (ethylenediaminetetraacetic acid disodium) is employed to seize *h*^+^ during photocatalytic processes [81,82]. 

**Figure 26:**
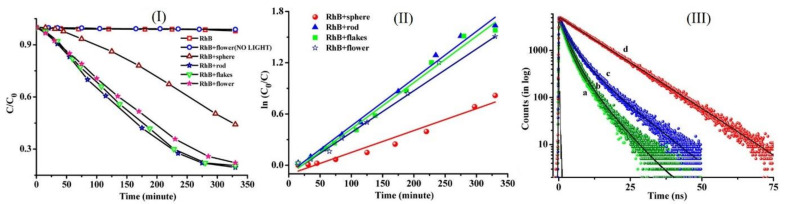
(**I**) Morphology-dependent photocatalytic activity of different H_2_TCPP aggregates; (**II**) degradation kinetics; (**III**) fluorescence decay plots: (a) nanosphere, (b) nanorod, (c) nanoflower, and (d) monomeric H_2_TCPP. Adapted from Ref. [68].

In one example, various morphologies of H_2_TCPP showed different efficiencies for the degradation of water-soluble RhB (Figure 13) [68]. The photodegradation rates were estimated as 56% (nanospheres), 71% (nanoflowers), 79% (nanoflakes), and 81% (nanorods) (Figure 26). Fluorescence decay data revealed that the H_2_TCPP molecules in the nanoflakes and nanoflowers exhibited both *J*- and *H*-aggregations. In contrast, in the case of the nanorod aggregates, the H_2_TCPP molecules showed only *J*-aggregation, which increased the electronic delocalization of charge carriers and facilitated the degradation reaction (Figure 26). The *J*-aggregated structures improved coherent electronic delocalization due to the strong intermolecular π electronic coupling that occurred between the coherently aligned chromophores and became efficient photosemiconductors. 

**Figure 27:**
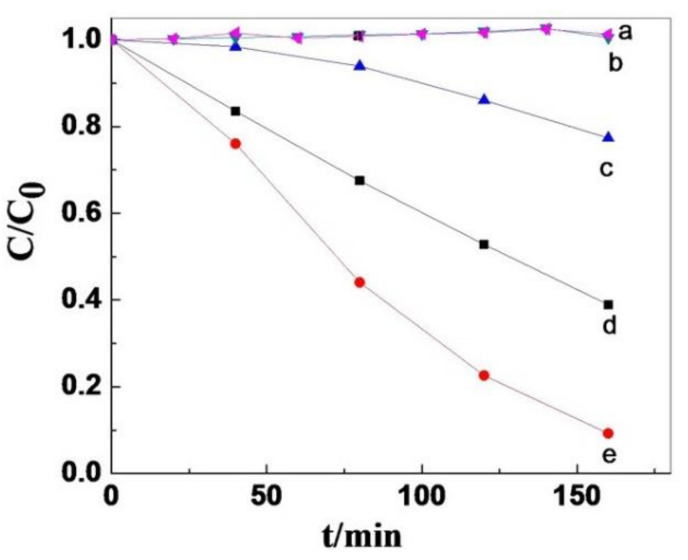
Photodegradation of MO dye by various SnCl_2_TPP nanostructures. No catalyst (**a**), TiO_2_ (P-25) (**b**), nanosheets (**c**), octahedra (**d**), and nanospheres (**e**). Adapted from Ref. [69].

In another study, SnCl_2_TPP nanocrystals exhibited attractive morphology-dependent photocatalytic performance for the decomposition of MO dye in the presence of sunlight (Figure 14) [69]. The tin porphyrin nanosheets exhibited 20% MO degradation after 160 min. In contrast, the photodegradation performance of MO exceeded 60% for nanooctahedra and 90% for nanospheres (Figure 27). 

**Figure 28:**
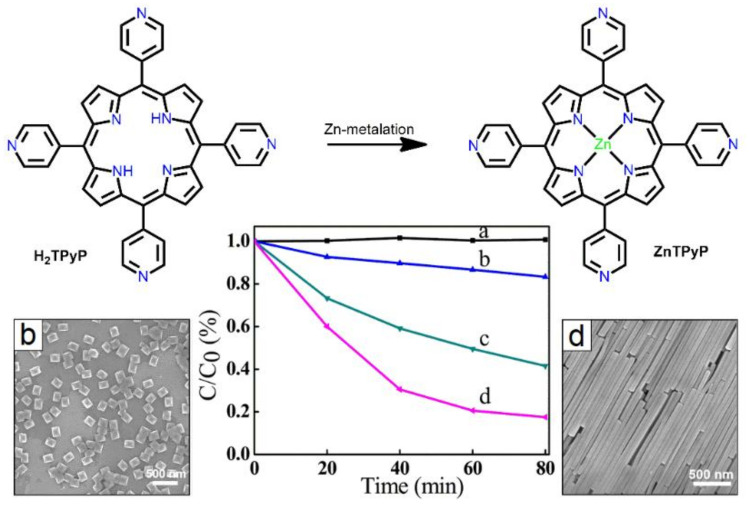
Photodegradation of MO dye by various nanostructures. No catalyst (**a**), (**b**) H_2_TPyP nano octahedra. (**c**) Intermediate nanoparticles. (**d**) ZnTPyP nanowires. Adapted from Ref. [83].

Fan et al. reported on nanostructures derived from H_2_TPyP and ZnTPyP via surfactant-assisted reprecipitation [83]. The morphologies varied from nanooctahedra for H_2_TPyP to nanowires for ZnTPyP. By attentively controlling the assembly behavior, ZnTPyP nanowires exhibited higher photocatalytic performance towards MO dye than H_2_TPyP nanooctahedra (Figure 28). 

**Figure 29:**
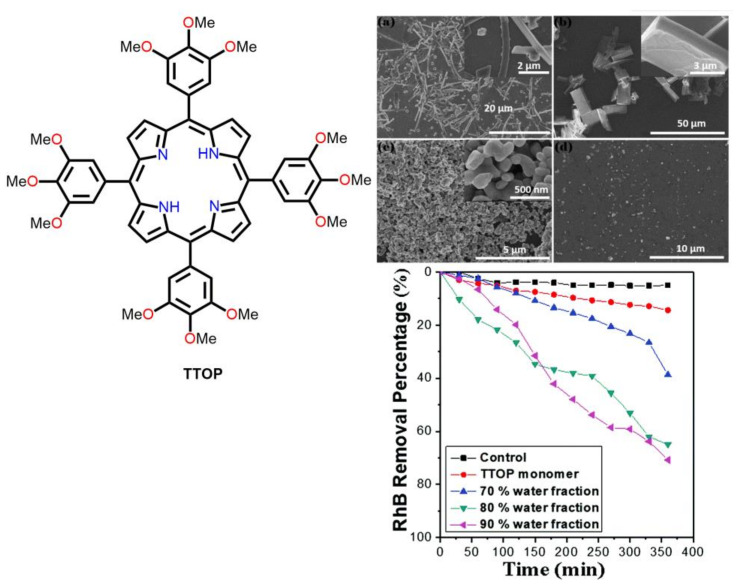
Photodegradation of RhB dye by different morphologies of TTOP fabricated using THF with different H_2_O fractions of 70% (**a**), 80% (**b**), 90% (**c**), and 95% (**d**). Adapted from Ref. [84].

Bhosale et al. reported on the morphology-dependent photodegradation of RhB using TTOP nanostructures [84]. The dimensions of the nanoaggregates could be regulated simply by adjusting the THF/H_2_O ratio. The photodegradation rate constant of the RhB dye was observed to be 0.0043 min^−1^ for the TTOP monomer. However, the photodegradation rate constants of the RhB dye for the aggregates fabricated with the THF/H_2_O ratio were found to be 0.678 h^−1^ for THF/H_2_O (3:7), 0.156 h^−1^ for THF/H_2_O (2:8), and 0.21 h^−1^ THF/H_2_O (1:9) (Figure 29). 

**Figure 30:**
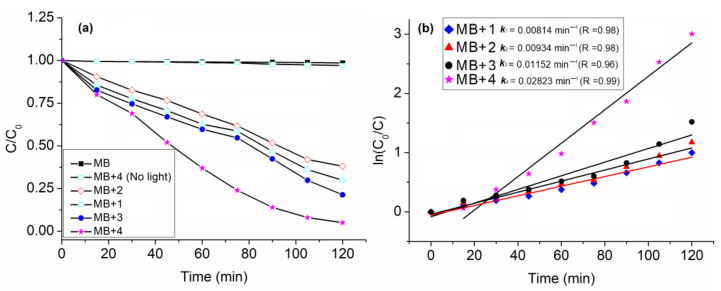
Photocatalytic degradation of MB dye by triads with different morphologies in the presence of visible light (**a**) and first-order rate kinetics (**b**). Adapted from Ref. [75].

Kim et al. reported on the morphology-controlled photodegradation of MB using Sn(IV)porphyrin-based nanomaterials (Figure 20) [75]. The photodegradation rate of the MB dye obtained when using nanofibers derived from triad **4** (1.68 h^−1^) was higher than that obtained when using nanocomposites from triad **3** (0.67 h^−1^), nanoparticles from triad **2** (0.54 h^−1^), and nanocomposites from triad **1** (0.48 h^−1^) (Figure 30). These observations suggest that the catalytic photodegradation performance of the MO dye was affected by the morphology of the photocatalyst. It is likely that different morphologies can affect catalytic activity because they provide different shapes, permanent porosities, surface energies, and numbers of active and defect sites. Moreover, strong electronic delocalization of the photogenerated charged species occurred on the surface of the catalysts. This improves the delay in the recombination of electron–hole pairs and thus results in photodegradation activity. Overall, the morphology of the catalyst controls the charge-transfer process and photocatalytic activity. 

**Figure 31:**
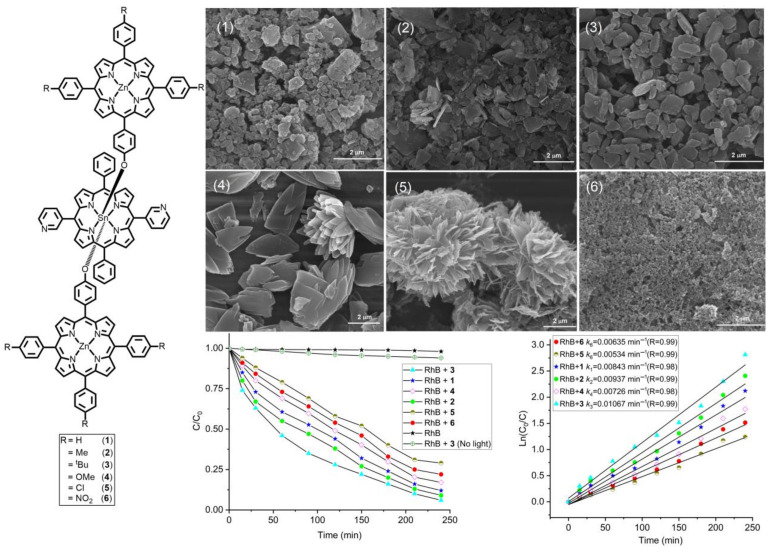
Photodegradation of RhB dye by various morphologies obtained from metalloporphyrin-based triads under visible-light irradiation. T1 (**1**), T2 (**2**), T3 (**3**), T4 (**4**), T5 (**5**), and T6 (**6**). Adapted from Ref. [85].

In another study, Kim et al. reported the morphology-dependent photodegradation of RhB in various nanoaggregates derived from metalloporphyrin-based triads [85]. These triads exhibited divergent nanoaggregates because of the various side chains found in the axial zinc porphyrin segments. The RhB dye degradation rate constant (first order) of T3 (1.0 × 10^−2^ min^−1^) was slightly higher than that of T1 (4.8 h^−1^), T2 (0.54 h^−1^), T4 (0.42 h^−1^), T5 (0.30 h^−1^), and T6 (0.36 h^−1^) (Figure 31).

**Figure 32:**
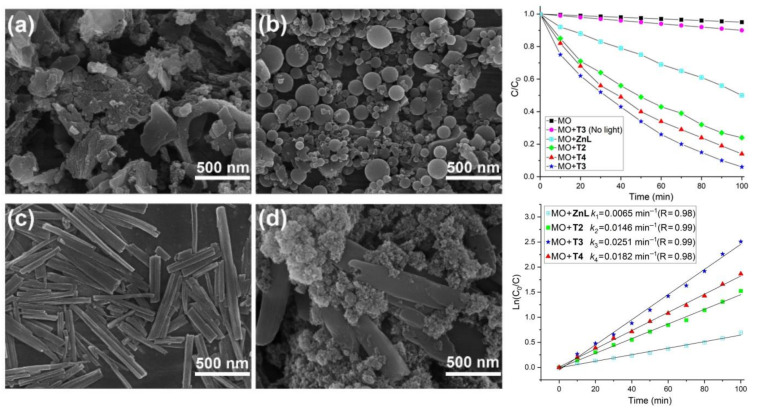
Morphology-controlled degradation of MO dye by several metalloporphyrin-based triads under visible-light irradiation. ZnL (**a**), T2 (**b**), T3 (**c**), and T4 (**d**). Adapted from Ref. [51].

The same group observed the morphology-dependent photodegradation of MO using metalloporphyrin-based triads under sunlight [51]. The reaction of ZnL (*meso*-5-(4-hydroxyphenyl)-10,15,20-tris(4-octoxyphenyl)porphyrinato)zinc(II)) with *trans*-dihydroxo-[5,15-bis(2-pyridyl)-10,20-bis(phenyl)porphyrinato] tin(IV), *trans*-dihydroxo-[5,15-bis(3-pyridyl)-10,20-bis(phenyl)porphyrinato] tin(IV), and *trans*-dihydroxo-[5,15-bis(4-pyridyl)-10,20-bis(phenyl)porphyrinato] tin(IV) in anhydrous toluene under an Ar atmosphere led to the formation of T2, T3, and T4 respectively. The positions of the pyridyl nitrogen atoms in the tin(IV)porphyrin building blocks differed. The photodegradation rate of MO was calculated to be 76–94% under sunlight within 100 min. Nanoflake-shaped T4 and nanosphere-shaped T2 exhibited lower catalytic performances than nanorod-shaped T3 (Figure 32). It is likely that porphyrin nanostructures increase solar light absorption, owing to the formation of aggregated architectures as compared to monomeric units. Extensive delocalization occurs on the surface of the nanostructures through the *π*-*π* interactions. This lowers charge recombination by stabilizing the photogenerated electron–hole pairs, subsequently enhancing lifetime and improving degradation performance. Therefore, morphology controls the photocatalytic activity of porphyrin-based nanostructures.

**Figure 33:**
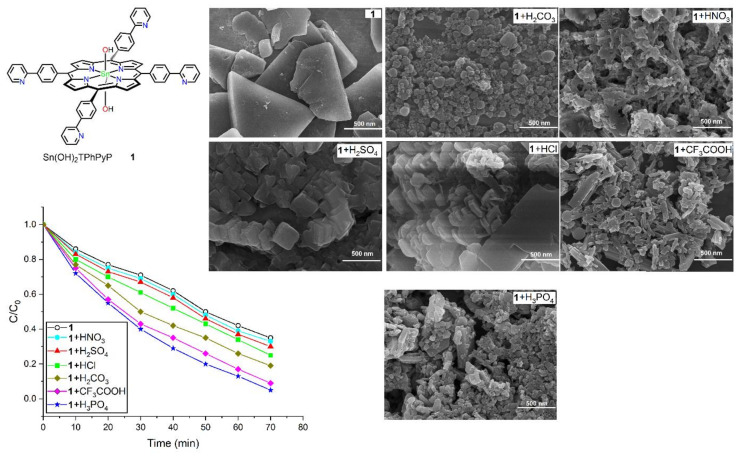
Morphology-controlled photodegradation of MG dye by various ionic complexes. Adapted from Ref. [86].

Kim et al. synthesized several tin-porphyrin-containing ionic compounds by reacting several acids (HCl, HNO_3_, H_2_SO_4_, H_2_CO_3_, CF_3_COOH, and H_3_PO_4_) with Sn(OH)_2_TPhPyP (**1**) [86]. These ionic complexes showed morphology-dependent photocatalytic activity against the decay of MG dye in sunlight, with efficiencies ranging from 50% to 95% (Figure 33) in an aqueous solution. The rate of the photodegradation of MG dye by photocatalyst **1** was 0.84 h^−1^. However, the rate constants of the ionic compounds obtained from the reaction of photocatalyst **1** with HNO_3_, HCl, H_2_SO_4_, H_2_CO_3_, CF_3_COOH, and H_3_PO_4_ were 0.9 h^−1^, 1.08 h^−1^, 0.96 h^−1^, 1.32 h^−1^, 1.92 h^−1^, and 2.34 h^−1^, respectively.

**Figure 34:**
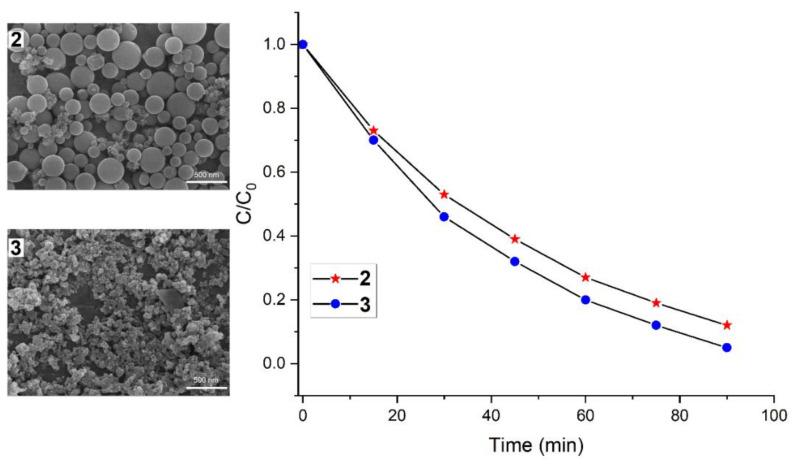
Morphology-controlled photodegradation of EBT dye by **2** and **3**. Adapted from Ref. [87].

The same group reported two porphyrin-based molecular square arrays: **2** and **3** [76]. These compounds were constructed from the 72 h refluxing of Re(CO)_5_Cl in mixed solvents (THF + toluene) with trans-dihydroxo-[5,15-bis(4-pyridyl)-10,20-bis(phenyl)porphyrinato]tin(IV) and trans-dihydroxo-[5,15-bis(4-pyridyl)-10,20-bis(4-tert-butylphenyl)-porphyrinato]tin(IV), respectively [87]. The structural configuration of these frameworks was rigid, owing to the locked 2D tetrameric array. Changing the side chain on the metalloporphyrin building blocks not only generated structural motifs but also led to the formation of precisely divergent nanoaggregates. These non-aggregated materials showed effective photodegradation of EBT dye in sunlight. The removal efficiencies were 88% for molecular array **2** and 95% for molecular array **3** within 90 min. It was observed that the photodegradation rate of EBT dye obtained when using molecular array **3** (1.92 h^−1^) was higher than that obtained when using molecular array **2** (1.38 h^−1^) (Figure 34).

## 4. Future Perspectives

Thus far, our discussion has been limited to self-assembled nanomaterials fabricated from various porphyrin derivatives and has examined the photocatalysis of pollutant dyes as free-standing organic semiconductors. During the photocatalysis process, photoinduced charge pairs (*e*^−^ and *h*^+^) experience either recombination or reach the interface of the photocatalyst and facilitate several photodegradation reactions. To improve photocatalytic performance in terms of surface area, light-harvesting capacity, reusability, and coherent charge separation, porphyrins or nanostructured porphyrins can be combined with other nanomaterials, such as various organic (graphene, graphene oxide, or reduced graphene oxide) or inorganic semiconductors (TiO_2_, ZnO, g-C_3_N_4_, etc.).

**Figure 35:**
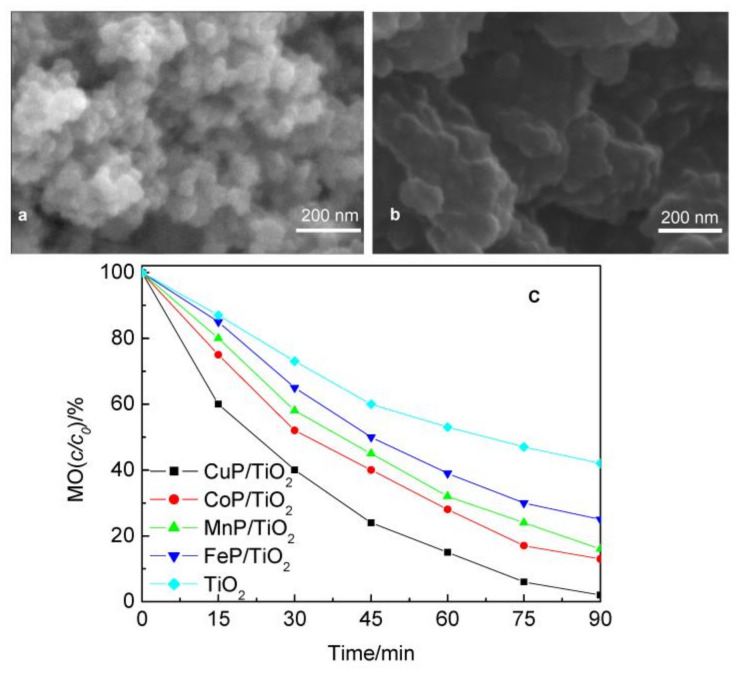
Morphology of TiO_2_ (**a**) and CuP-TiO_2_ (**b**). Photocatalytic degradation of MO dye (**c**). Adapted from Ref. [88].

Inorganic semiconductors such as TiO_2_ can be integrated with metalloporphyrin aggregates to permit light accumulation in a broad range of sunlight. This improves charge dissociation and photodegradation efficiency. Ji et al. demonstrated the improved photodegradation activity of various metalated (Cu, Mn, Fe, and Co) complexes of *meso*-tetraphenylporphyrin adsorbed on the surface of TiO_2_ (P-25) [88]. It should be mentioned that copper porphyrin-immobilized TiO_2_ (CuP-TiO_2_) exhibited outstanding photodegradation performance for the decay of MO over other metalloporphyrins under UV light irradiation (Figure 35). The high photocatalytic activity of CuP-TiO_2_ can be explained by the fact that Cu^2+^ readily gains an electron to reach a steady state, causing the isolation of electrons with holes compared to other metal ions in the composites. Moreover, Cu^2+^ (0.73 Å) and Ti^4+^ (0.64 Å) have similar ionic radii. Therefore, Cu^2+^ can easily penetrate TiO_2_ as a deep acceptor in conjunction with the neighboring O vacancies of Ti^4+^.

**Figure 36:**
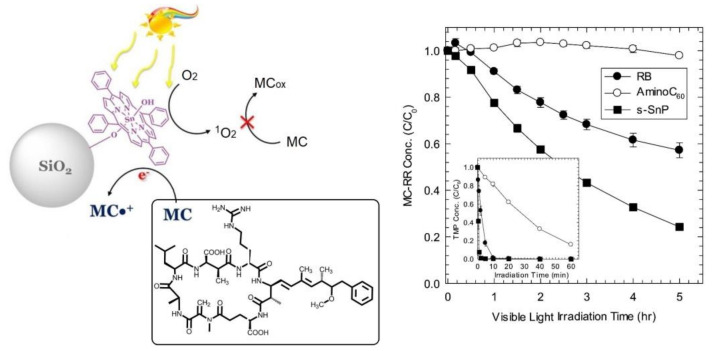
Photochemical degradation of RR-MC by silica/SnP-based photosensitizers (inset: photodegradation of 2,4,6-trimethylphenol) and heterogeneous sanitizers (RB, aminoC_60_, s-SnP). Reproduced from Ref. [89].

Lee et al. demonstrated that silica-immobilized tin porphyrin (silica/SnP or s-SnP) photosensitizers facilitate the degradation of microcystins (MCs) despite the low generation of photosensitized singlet oxygen [89]. An equivalent study using Rose Bengal (RB), C60 aminofullerene, and s-SnP suggested that the RR-MC decomposition rate is directly related to the photosensitizing properties (Figure 36). This study also suggested that direct electron movement from MCs to the triplet state of SnP facilitates the photodegradation of MCs under visible-light irradiation. Moreover, the incorporation of tin porphyrin not only facilitates direct photo-initiated electron transfer but also creates large surface modifications for effective MC sorption on the surface of hybrid catalysts. Singlet oxygen is no longer a reactive species in the degradation of MCs. This was confirmed by the negligible effects of singlet oxygen scavengers such as azide and D_2_O on the MC-RR degradation rate.

**Figure 37:**
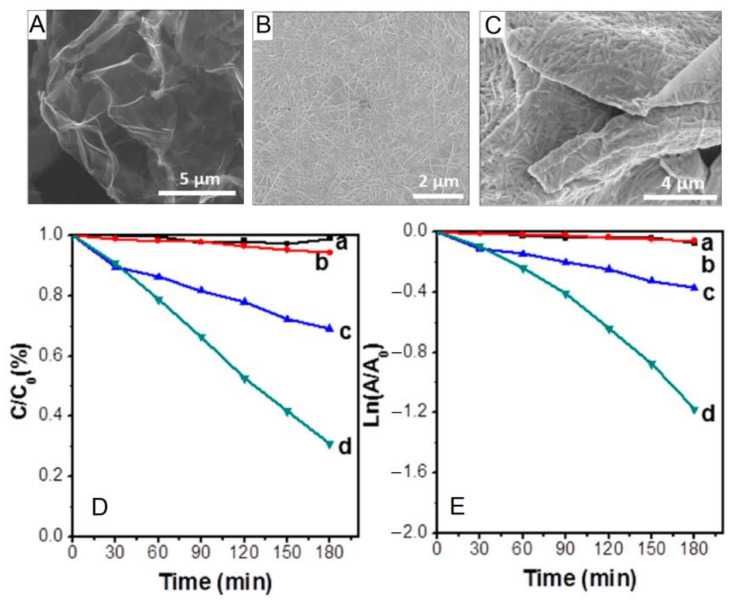
Morphology (SEM) of (**A**) GNPs, (**B**) free-standing H_2_TCPP nanoaggregates, and (**C**) GNPs@H_2_TCPP. Photocatalytic performance (**D**) and kinetic simulation curve (**E**) for MO dye; (a) blank, (b) GNPs, (c) H_2_TCPP nanoaggregates (free-standing), (d) GNPs@H_2_TCPP. Adapted from Ref. [90].

Graphene nanoparticles (GNPs) are one of the most common types of carbon-based compounds that are integrated with the porphyrin nanostructure due to their excess electron mobility and large surface area. During the photocatalytic reaction, graphene effectively transfers the photogenerated reactive pairs to the adsorbed dyes on the surface and enhances the degradation rate. La and co-authors demonstrated the fabrication of a graphene-incorporated porphyrin nanofiber (GNPs@H_2_TCPP). With the support of D-arginine, self-assembled H_2_TCPP was fabricated on the surface of graphene nanoparticles accompanied by the fabrication of GNPs@H_2_TCPP [90]. It was observed that this hybrid nanomaterial showed remarkable photocatalytic degradation performance against methyl orange dye (removal rate ~80%) after 180 min of visible-light irradiation (Figure 37). This could be attributed to graphene increasing the lifetime of the charged pairs by preventing the recombination of photogenerated reactive pairs generated by the porphyrin nanofiber. 

**Figure 38:**
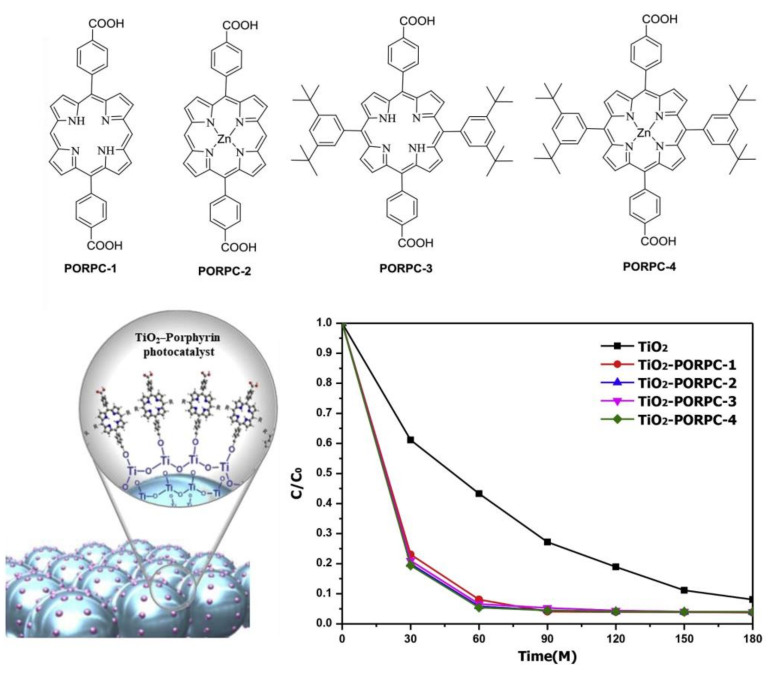
Various TiO_2_-porphyrin photocatalysts for photodegradation of MB dye. Adapted from Ref. [91].

Min et al. demonstrated that effective binding between porphyrinoids and optoelectronic materials is important for improving the photodegradation of toxic dyes. To this end, they synthesized different heterocomplexes based on the carboxyphenyl side chains of various porphyrins and anatase TiO_2_ [91]. All these hybrid materials exhibited enhanced photodegradation performance compared to pure TiO_2_ (Figure 38). The photodegradation activity of MB dye was 95%, compared to only 55% for pure TiO_2_, in the presence of sunlight within 1 h. Additionally, the authors observed that zinc-metalated porphyrins showed higher photocatalytic performance than the metal-free carboxyphenyl derivatives of porphyrins. This may be due to the electronic coupling between the zinc carboxyphenyl derivatives of the porphyrins and the CB of TiO_2_. Therefore, chemical surface modification with carboxyphenyl porphyrin and TiO_2_ not only strengthened the bonds between them but also improved the photocatalytic performance and prevented the separation of porphyrins from the TiO_2_ surface.

**Figure 39:**
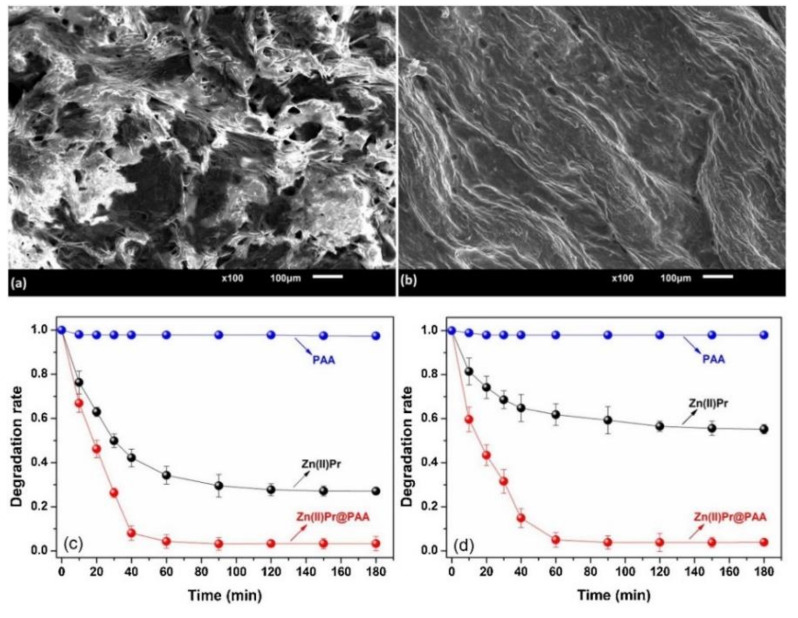
Morphology (SEM) of (**a**) PAA and (**b**) Zn(II)Pr@PAA. Photodegradation of (**c**) MB and (**d**) NB. Adapted from Ref. [92].

Fajardo et al. reported a hybrid microparticle (Zn(II)Pr@PAA) for the photodegradation of toxic pollutants under sunlight (where PAA = polyacrylic acid) [92]. Zn(II)-porphyrin was easily incorporated into the PAA matrix in situ to form Zn(II)Pr@PAA. Various analytical and microscopic methods confirmed that the incorporated metalloporphyrin influenced the optical properties of the Zn(II)Pr@PAA microstructures. The resulting photocatalysts exhibited improved photocatalytic performance with respect to the decomposition of nitrobenzene (NB) and MB dyes compared to the starting Zn(II)porphyrin (Figure 39). The degradation rate constants of all organic pollutants varied from 0.03 to 0.05 min^−1^.

**Figure 40:**
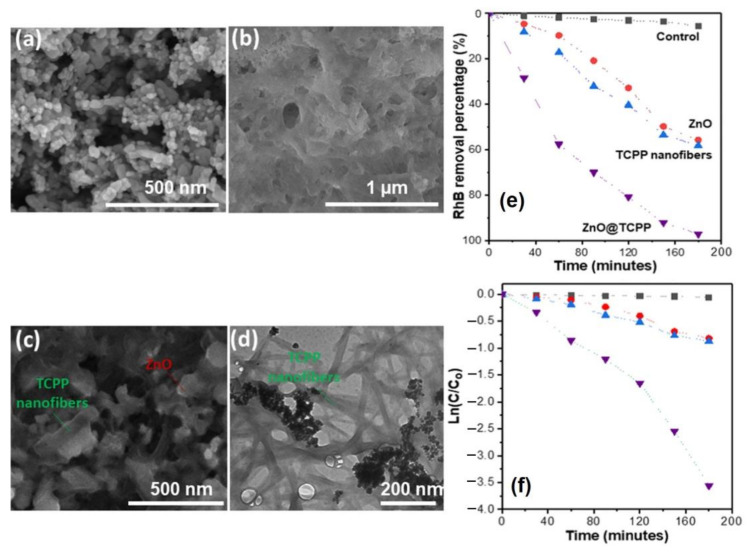
Morphology of ZnO-NPs: (**a**) SEM, (**c**) TEM. Morphology of ZnO@H_2_TCPP nanofibers: (**b**) SEM, (**d**) TEM. (**e**) Degradation of RhB and (**f**) kinetic study. Adapted from Ref. [93].

Vo et al. demonstrated the improved photodegradation activity of ZnO@H_2_TCPP nanofibers [93]. The ZnO@H_2_TCPP nanofiber hybrid complex was forged via reprecipitation self-assembly. The results confirmed the consistent incorporation of the ZnO nanoparticles (ZnO-NPs) into the self-assembled H_2_TCPP nanofiber matrix. The bandgap energies of the TCPP nanofibers (2.6 eV), ZnO nanoparticles (3.21 eV), and ZnO@H_2_TCPP (3.12 and 2.43 eV) were determined using the Tauc plot method. The fabrication of porphyrin nanofibers on the ZnO nanoparticles lowered the bandgap energy and enhanced their light-harvesting properties. Due to the synergetic effect of the ZnO and H_2_TCPP nanofibers, the ZnO@H_2_TCPP composite showed outstanding photocatalytic activity for RhB degradation (rate constant = 0.02 min^−1^). Approximately 97% of the dye was removed after 180 min of visible-light irradiation (Figure 40).

**Figure 41:**
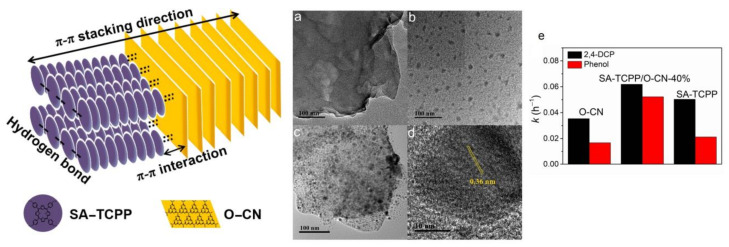
Spatial conformation of SA-TCPP/O-CN. Morphology (TEM) of O-CN (**a**), SA-TCPP (**b**), and SA-TCPP/O-CN-40% (**c**). HRTEM of SA-TCPP/O-CN-40% (**d**). (**e**) Photodegradation rate constants of organic pollutants. Adapted from Ref. [94].

Xu et al. fabricated a hybrid porphyrin-based heterojunction (SA-TCPP/O-CN) incorporated in situ via the reaction of H_2_TCPP nanoaggregates (SA-TCPP) and oxygen-doped g-C_3_N_4_ (O-CN) nanosheets [94]. In TEM and HRTEM studies, it was observed that O-CN exhibited a nanosheet shape with a length of a few hundred nanometers. In contrast, SA-TCPP showed nanocrystals, with an average diameter varying from 5 to 20 nm. It was found that the self-assembled porphyrin nanoaggregates were regularly fabricated on the surface of oxygen-doped g-C_3_N_4_ in SA-TCPP/O-CN, confirming the construction of a 0D/2D assembled hybrid structure. The formed 0D/2D heterostructures were successfully assembled via π-π interactions. The homogeneous fabrication of SA-TCPP/O-CN facilitated electron delocalization and promoted interfacial charge transfer. The energy-harvesting property of O-CN was significantly enhanced by the wide-spectrum reactive capacity of SA-TCPP. O-CN and SA-TCPP synergistically contributed to the enhanced photodegradation of phenol and 2,4-dinitrophenol (Figure 41). 

**Figure 42:**
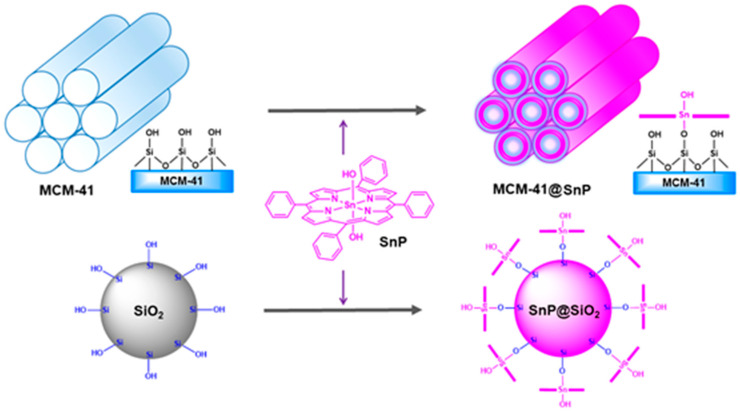
Synthesis scheme for SiO_2_@SnP and MCM-41@SnP. Adapted from Ref. [95].

Recently, Kim et al. fabricated two heterostructures (MCM-41@SnP and SiO_2_@SnP) derived from the chemisorption of SnP on SiO_2_ nanoparticles and mesoporous structured MCM-41 [95]. The immobilization of SnP on SiO_2_ and MCM-41 not only increased the surface area but also modified the morphology of the composites. These factors facilitated the photodegradation of RhB, erioglaucine, and m-cresol purple dyes in the presence of sunlight. The photodegradation rates of these dyes obtained when using the abovementioned photocatalysts varied from 87 to 95% under sunlight. MCM-41@SnP showed higher photocatalytic activity towards the decay of pollutant dyes than SiO_2_@SnP and SnP. MCM-41@SnP exhibited outstanding photostability and reusability (Figure 42). 

**Figure 43:**
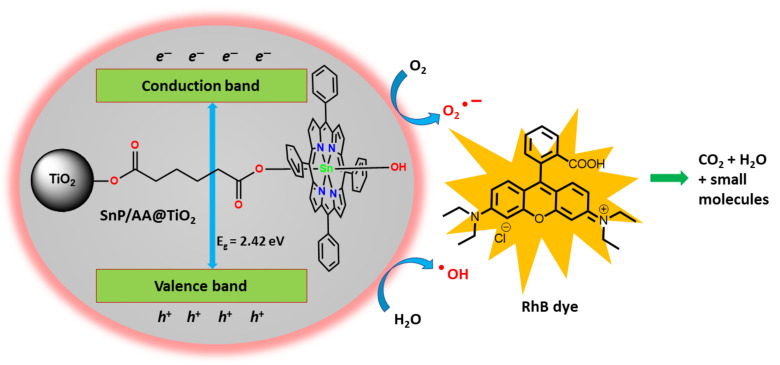
Surface-modified photocatalyst **4** for photocatalytic degradation of RhB dye. Adapted from Ref. [96].

In another study, the same group reported an efficient nanomaterial, SnP/AA@TiO_2_ (photocatalyst **4**, AA = adipic acid), constructed by exploiting the coordination interaction between AA and the axial hydroxyl group in SnP on the TiO_2_ surface [96]. The SnP molecule was firmly attached to the TiO_2_ surface through an AA interconnection in photocatalyst **4**, as verified by several spectroscopic techniques. Under visible-light irradiation, photocatalyst **4** exhibited outstanding photocatalytic RhB degradation. Photocatalyst **4** achieved 95% RhB dye removal within 80 min. The first-order decomposition rate constant was observed to be 3.66 × 10^−2^ min^−1^. The high degradation rate and greater stability of SnP-anchored photocatalysts make them more coherent than SnP@TiO_2_ or TiO_2_ (Figure 43). 

**Figure 44:**
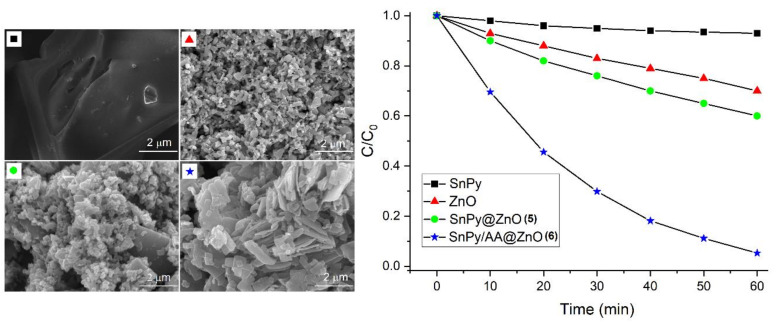
Surface-modified photocatalysts for photodegradation of AM dye. Adapted from Ref. [97].

Kim et al. reported two hybrid catalysts, SnPy@ZnO (**5**) and SnPy/AA@ZnO (**6**). The reaction of ZnO with *trans*-dihydroxo [5,10,15,20-tetrakis(4-pyridyl)porphyrinato]tin(IV) (SnPy) led to the formation of photocatalyst **5**. Photocatalyst **6** was prepared via the reaction of ZnO nanoparticles with pretreated adipic acid (AA) [97]. In the case of photocatalyst **5**, ZnO and SnPy were probably connected by a coordinative interaction between the Zn atom of the ZnO surface and the pyridyl-N atom of SnPy. In contrast, in the case of photocatalyst **6**, the SnPy molecule was firmly combined with the ZnO nanostructure via adipic acid anchors. Photocatalyst **6** showed significantly improved photodegradation performance for the decay of AM dye under sunlight compared to SnPy, ZnO, and photocatalyst **5**. The first-order photodegradation rates of AM dye were observed to be 0.06 h^−1^ (SnPy), 0.036 h^−1^ (ZnO), 0.048 h^−1^ (photocatalyst **5**), and 2.88 h^−1^ (photocatalyst **6**) (Figure 44).

Surface modification not only enhances the delocalization of the excited state of photogenerated electron–hole pairs and improves photocatalytic performance, but it also results in the generation of active sites for pollutants that are larger than those of TiO_2_, ZnO, or SnP. 

## 5. Conclusions

This article provides an overview of the significant progress made in porphyrin-based nanomaterials for the photodegradation of contaminants under sunlight. Porphyrin-based self-assembled nanostructures are constructed using several methods, such as ionic self-assembly, reprecipitation, sonication-assisted, metal–ligand coordination, and surfactant-assisted methods. Various interactions, including π-π interactions, hydrogen bonding interactions, coordination bonding interactions, and electrostatic interactions, are commonly involved in the self-assembly of porphyrin molecules to fabricate nanostructures in a solid state or solution. All these porphyrin nanomaterials show remarkable photocatalytic performance compared to monomeric amorphous porphyrins for the removal of toxic pollutants under visible-light irradiation. This is because porphyrin nanoaggregates with ordered molecular structures enhance charge separation compared with monomeric porphyrins. The synthetic utility of porphyrins permits the organization of porphyrin building blocks into nanostructures, which can remarkably improve their light-harvesting properties and photostability. The tunable functionalization and distinctive structure of porphyrin nanomaterials trigger the junction of the charge-transfer mechanism and facilitate the photodegradation of pollutant dyes. However, despite the advances made in integrated methodologies to fabricate various porphyrin-based nano- or micro-structures, the photodegradation activity of these systems is still limited by the rapid recombination of photogenerated reactive pairs. Various porphyrin nanoaggregates have been incorporated with other nanoparticles, including graphene oxide, TiO_2_, ZnO, and g-C_3_N_4_, to enhance photodegradation activity in terms of light-harvesting capacity, permanent porosity, recyclability, and significant charge separation. Therefore, a composite of optoelectronic functional materials and porphyrins can contribute to the fabrication of nanohybrid materials for visible-light photocatalysis. Further investigation of porphyrin nanomaterials or semiconductor/porphyrin composites is required to improve their photocatalytic efficiencies. Thus far, discussions have been limited to the photodegradation of pollutants in water. The fabrication of novel porphyrin-based nanoaggregates in conjunction with the high harvesting efficiency of solar light could also be a major point for future research and applications in many important areas. The advantages of porphyrin-based nanoaggregates have been exploited in several important areas, such as fuel cells [98], sensing [99], H_2_ production [100], biomedical applications [101], CO_2_ reduction [102], cancer treatment [103], catalysis [104], and gas storage and separation [105]. 

In conclusion, we demonstrated that porphyrin-based nanomaterials acquired via self-assembly are ideal photocatalytic compounds for harvesting solar energy from a wide range of light spectra. These nanoaggregates are easily fabricated and modified, and they exhibit high photodegradation performance. Their capacity to absorb solar energy in the visible region and their effective electron transfer properties make them unique compared to metal oxide nanoparticles. Versatile porphyrin derivatives conjugated with various semiconducting materials will likely expand the degradation performance of environmentally friendly, low-cost, robust, and highly recyclable photocatalysts for water treatment in the future.

## Data Availability

Not applicable.

## Abbreviations

AOP	Advanced oxidation process
AM	Amaranth
CB	Conduction band
CTAB	Cetyltrimethylammonium bromide
DCM	Dichloromethane
DASS	1-Decanesulfonic acid sodium salt
DLS	Dynamic light scattering
DMF	*N*, *N*-dimethylformamide
EBT	Eriochrome Black T
Eg	Band gap energy
H_2_TCPP	5,10,15,20-Tetrakis(4-carboxyphenyl) porphyrin
H_2_TPyP	5,10,15,20-Tetrakis(4-pyridyl)porphyrin
H_2_THPP	5,10,15,20-Tetrakis(4-(hydroxyl)phenyl)porphyrin
H_4_TPPS_4_^2−^	5,10,15,20-Tetrakis(sulfonatophenyl)porphyrin
MeOH	Methanol
MB	Methylene blue
MO	Methyl orange
MCM-41	Mobil Composition of Matter No. 41
RhB	Rhodamine B
ROS	Reactive oxygen species
SEM	Scanning electron microscopy
SnCl_2_TPP	Dichloro-(5,10,15,20-tetraphenylporphyrinato)tin(IV)
SnP	*trans*-Dihydroxo(5,10,15,20-tetraphenylporphyrinato)tin(IV)
TEM	Transmission electron microscopy
THF	Tetrahydrofuran
VB	Valence band
XRD	X-ray diffraction
ZnTPyP	[5,10,15,20-Tetrakis(4-pyridyl)porphyrinato]zinc(II)
